# Phylogenomic analyses and chromosome ploidy identification reveal multiple cryptic species in *Allium sikkimense* complex (Amaryllidaceae)

**DOI:** 10.3389/fpls.2023.1268546

**Published:** 2024-01-04

**Authors:** De-Qing Huang, Xiang-Guang Ma, Hang Sun

**Affiliations:** ^1^ College of Pharmacy, Guilin Medical University, Guilin, China; ^2^ CAS Key Laboratory for Plant Diversity and Biogeography of East Asia, Kunming Institute of Botany, Chinese Academy of Sciences, Kunming, China

**Keywords:** *Allium sikkimense* complex, budding speciation, cryptic species, ecological divergence, genome size, Hengduan Mountains, phylogeny, polyploidy

## Abstract

Polyploidization is a process that typically leads to instantaneous reproductive isolation and has, therefore, been considered as one of the major evolutionary forces in the species-rich Hengduan Mountains (HM), yet this topic remains poorly studied in the region. *Allium sikkimense* and its relatives (about eight species) compose a natural diploid–polyploid complex with the highest diversity in the HM and adjacent areas. A combination of nuclear ribosomal DNA (nrDNA), plastome, transcriptome, and ploidy identification through chromosome counting and flow cytometry is employed to reconstruct the phylogenetic relationships in this complex and to investigate the frequency and the evolutionary significance of polyploidy in the complex. The plastome failed to resolve the phylogenetic relationships of the different species in the *A. sikkimense* complex, and the phylogenetic tree based on nrDNA also has limited resolution. However, our study reveals a well-resolved phylogenetic framework for species in the *A. sikkimense* complex using more than 1,000 orthologous genes from the transcriptome data. Previously recognized morphospecies *A. sikkimense* are non-monophyletic and comprise at least two independently evolved lineages (i.e., cryptic species), each forming a clade with different diploid species in this complex. The embedded pattern of octoploid *A. jichouense* and tetraploid *A.* sp. nov. within different polyploid samples of *A. sikkimense* supports a possible scenario of budding speciation (via niche divergence). Furthermore, our results reveal that co-occurring species in the *A. sikkimense* complex usually have different ploidy levels, suggesting that polyploidy is an important process for reproductive isolation of sympatric *Allium* species. Phylogenetic network analyses suggested that the phylogenetic relationships of the *A. sikkimense* complex, allowing for reticulation events, always fit the dataset better than a simple bifurcating tree. In addition, the included or exserted filaments, which have long been used to delimit species, are highly unreliable taxonomically due to their extensive parallel and convergent evolution.

## 1 Introduction

Polyploidy, also referred to as whole-genome duplication (WGD; i.e., polyploidization events), is now generally considered to be one of the main drivers of diversification (speciation) in vascular plants (Alix et al., 2017; Van de Peer et al., 2021), due to its widespread occurrence (34.5%) and frequent association with speciation events (approximately 15%) (Wood et al., 2009). Polyploids can originate from the same species (“autopolyploidy”) or from the hybridization of two different species (“allopolyploidy”). Compared to diploid ancestors, the increased genomic content of polyploidy potentially provides a strong tolerance to environmental stresses and increases the possibility to colonize new habitats (Levin, 1983; Otto and Whitton, 2000; Brochmann et al., 2004). Many studies have shown that polyploidy is positively correlated with species adaptation and diversification in flowering plants (Soltis et al., 2014; Van de Peer et al., 2017; Levin and Soltis, 2018; Ren et al., 2018; Han et al., 2020; Wu et al., 2022). Empirical evidence has shown that the occurrence of polyploidization events is closely associated with harsher environments (e.g., arid, arctic, and alpine habitats) and may enhance the ability of organisms to rapidly adapt to extreme environmental changes (Cai et al., 2019; Van de Peer et al., 2021). Polyploid species are not randomly distributed across the globe, with polyploid frequency increasing along a latitudinal gradient and towards higher latitudes (Rice et al., 2019). High mountain environments are also associated with climate variability and lower temperatures, which may lead to higher rates of WGD (Rice et al., 2019; Van de Peer et al., 2021; Wang et al., 2023).

The species-rich Hengduan Mountains is recognized as one of the world’s hot spots of biodiversity. The probable driving force for plant evolutionary radiations in this region may have resulted from the combined effects of (i) geographic isolation and divergent selection and (ii) hybridization (including homoploid hybrids but without genome duplication, interspecific introgression, and allopolyploidy) (Liu et al., 2014; Wen et al., 2014; Mao et al., 2021; Wu et al., 2022). Although previous studies have suggested that polyploidy may play a relatively minor role in the evolutionary diversification in this region (e.g., Nie et al., 2005), increasing evidence suggests that the contributions of auto- and allopolyploid events to species diversification may have been underestimated (Wu et al., 2022). For example, genetic divergence is not always accompanied by corresponding morphological changes, i.e., genetically or phylogenetically as independently evolved lineages or species, but morphologically indistinguishable (cryptic lineages/species) (Bickford et al., 2007). Cryptic polyploids are often difficult to detect (e.g., Ma et al., 2015), especially autopolyploids, which are often qualitatively morphologically cryptic.

Polyploidy shows a non-random distribution in different genera in the Hengduan Mountains, with some highly diversified taxa being unusually scarce, such as *Cremanthodium* (Liu et al., 2001), *Ligularia* (Liu, 2004), and *Delphinium* (Yuan and Yang, 2008), whereas some species-rich genera being common, e.g., *Anaphalis* (Meng et al., 2010), *Allium* (Han et al., 2020), *Meconopsis* (Yang et al., 2012), *Gentiana* (Chen et al., 2021a), and *Saxifraga* (Ebersbach et al., 2020). Previous studies have shown that the polyploid species in this region and adjacent areas may have undergone a very complex evolutionary history, e.g., multiple autopolyploidization, cryptic and repeated “allopolyploid” hybridization within *Allium przewalskianum* (Wu et al., 2010; Liang et al., 2015), and some extant polyploids even involving ghost species/lineages such as *Oxyria sinensis* (Luo et al., 2017) and *Allium tetraploideum* (Li et al., 2021). Therefore, polyploidization may also be one of the major evolutionary forces in the species diversity of the Hengduan Mountains, but this topic remains poorly studied so far (Wen et al., 2014; Mao et al., 2021; Wang et al., 2023).

*Allium* is a well-known example with high polyploid frequencies (almost 30.6%; Han et al., 2020), which is probably closely related to its biological traits, such as herbaceous life form (short generations), perenniality (sufficient time), asexual reproduction by clonal propagation (root sprouts of bulbs or rhizomes), and cold/drought tolerance, as these traits favor the repeated occurrence of polyploid and are also conducive to the maintenance of high polyploid potential (Van Drunen and Husband, 2019; Han et al., 2020). For this reason, previous studies have suggested that polyploidy has played an important role in the evolution and diversification of the genus *Allium* (Wu et al., 2010; Han et al., 2020; Li et al., 2021; Wang et al., 2022).

*Allium* sect. *Sikkimensia* (Traub) N. Friesen occurs predominantly in central-southwest China, with the highest diversity in the Hengduan Mountains and adjacent areas (e.g., Qinling-Daba Mountains) (Xie et al., 2019) and currently includes more than 13 described species (Huang et al., 2022). Of these, four species have been documented as diploid–polyploid complexes, particularly *A. sikkimense*, for which several different ploidy levels have been reported, including diploid, tetraploid, hexaploid, and aneuploid, and with different chromosome numbers ranging from 2n = 16 to 60 (**Supplementary Table S1**). In the case of *A. jichouense*, a newly proposed species of sect. *Sikkimensia* and characterized mainly by falcate or curled leaves, a significantly different genome size (corresponding to ploidy level) is detected compared to the sympatric *A. sikkimense* (Huang et al., 2022), indicating a potential polyploidization event. Accordingly, polyploidization may have led to the diversification and ecological specialization of *Allium* species in sect. *Sikkimensia*, making it an excellent model to study the influence of polyploidy on speciation and diversification.

The species-level phylogenetic relationships within sect. *Sikkimensia* (particularly the predominantly blue-flowered group) have been investigated using nuclear ribosomal DNA (nrDNA) and plastid DNA (ptDNA) fragments based on comparatively sufficient samples across the entire distribution range (Xie et al., 2019). Although the phylogenetic backbone of the blue-flowered group has been established (i.e., the phylogenetic hypothesis of four major lineages), deeper phylogenetic relationships between and within these lineages remain largely unclear. For example, the *A. sikkimense* clade [corresponding to lineage D of Xie et al. (2019) and Huang et al. (2022)] comprises three morphologically similar species, *A. sikkimense*, *A. beesianum*, and *A. yuanum*, with blue flowers and included filaments, and a superficially unrelated *A. plurifoliatum* var. *zhegushanense*, with completely different floral morphology (e.g., pale pink to light purple flowers and conspicuously exserted filaments) and habitat preference (under forests). The discordance between gene trees and morphology-based taxonomy has been attributed to plastid capture (via hybridization) and convergent evolution under similar environments (Xie et al., 2019). Lately, the newly reported species *A. jichouense* has also been shown to be nested within the *A. sikkimense* clade (Huang et al., 2022).

Because the species in the *A. sikkimense* clade are morphologically and phylogenetically related, we refer to them here as a species complex, the *A. sikkimense* complex (Sousa-Paula et al., 2021). The phylogenetic relationships among species within the *A. sikkimense* complex are not yet fully understood, particularly the nested phylogenetic status of other species in *A. sikkimense*. Whether this embedded phylogenetic scenario is due to the low phylogenetic power of the traditional genetic markers or to evolutionary processes such as polyploidization, hybridization, and incomplete lineage sorting (ILS), i.e., deep coalescence (ancestral gene copies fail to coalesce into a common ancestral copy until deeper than previous speciation events; Maddison, 1997). If this is not the case, then the embedded phylogenetic scenarios would lead to the currently accepted morphospecies *A. sikkimense* (identified only on the basis of morphological differences) being non-monophyletic, implying the existence of cryptic species within *A. sikkimense*. A more robust phylogenetic framework is necessary to address these questions in the *A. sikkimense* complex. Given the large genome size of *Allium*, transcriptome sequencing seems to be the best choice to obtain a large number of orthologous genes. The main objectives of this study are to use high-throughput sequencing data to reconstruct the phylogenetic relationships among the major lineages of the *A. sikkimense* complex in the context of sect. *Sikkimensia*, to investigate the occurrence of polyploidy within the *A. sikkimense* complex, to infer whether cryptic lineages or species exist within morphologically recognized *A. sikkimense*, and to gain insight into the influence of polyploidy on the diversification of the complex.

## 2 Materials and methods

### 2.1 Taxon sampling

**Figure 1:**
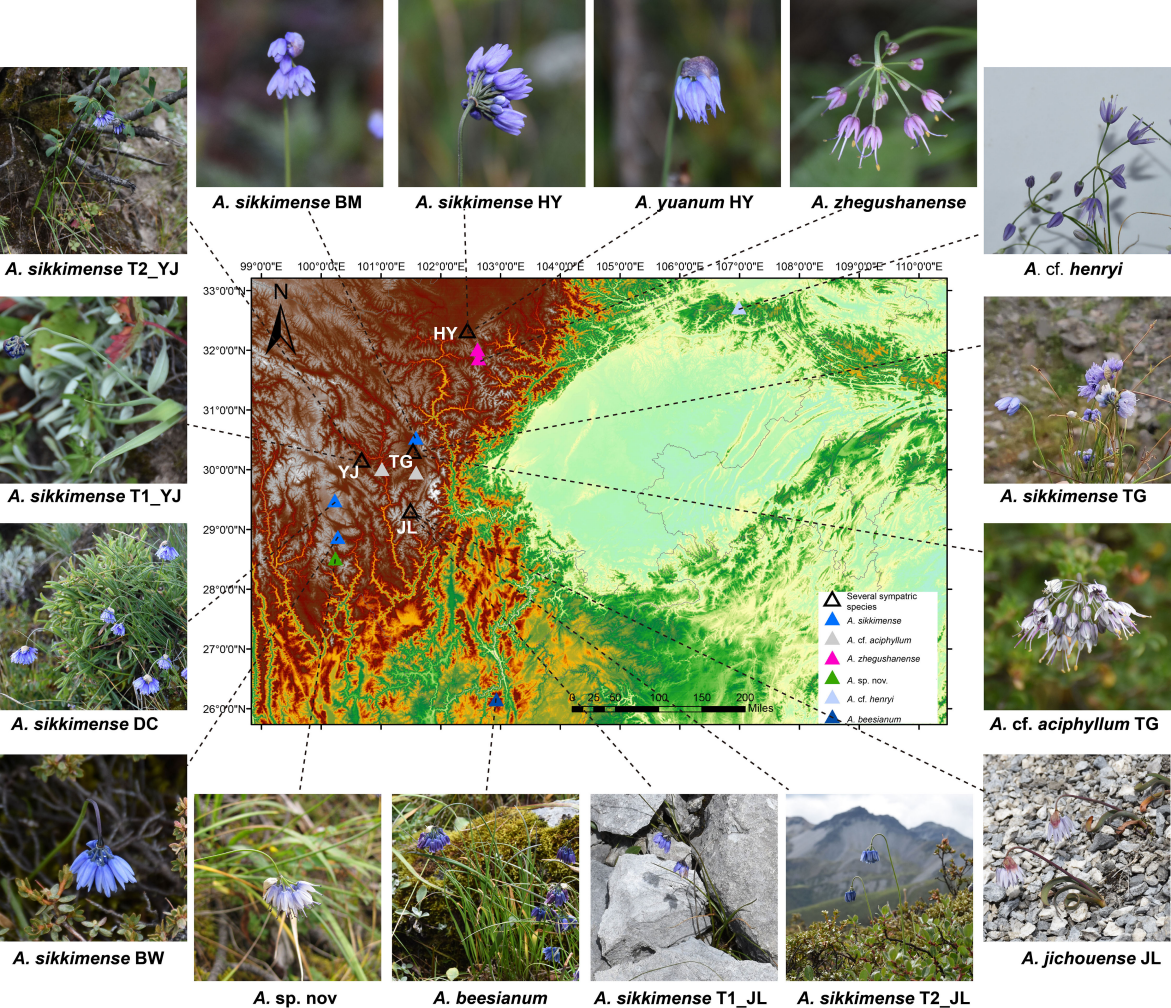
Map showing the distribution and morphology of the *A. sikkimense* complex sampled in this study.

Following the molecular phylogenetic framework of sect. *Sikkimensia* by Xie et al. (2019), we focus our taxon sampling mainly on the *A. sikkimense* complex in the Hengduan Mountains, which comprises about eight taxonomic units (*viz*., *A. beesianum*, *A.* cf. *aciphyllum*, *A.* cf. *henryi*, *A. jichouense*, *A. plurifoliatum* var. *zhegushanense*, *A. sikkimense*, *A.* sp. nov., and *A. yuanum*) and 38 individuals from 17 populations (**Table 1**). For *A.* sp. nov., which is morphologically similar to *A. cyaneum* (e.g., blue flowers and obviously exserted filaments) but phylogenetically more closely related to species of the *A. sikkimense* complex (see below) and is tentatively treated here as an undescribed species because only a few individuals from one population of Daocheng are available and more samples, especially from other populations, are needed to confirm the phylogenetic and taxonomic position of this species. The spatial distribution and morphological characteristics of the sampled populations are shown in **Figure 1**. Live plants were collected in the field, and fresh leaves were immediately frozen in liquid nitrogen for transcriptome sequencing and whole-genome re-sequencing. Dried leaves with silica gel were used for genome size measurements. Voucher specimens were deposited in the herbarium of the Kunming Institute of Botany, Chinese Academy of Sciences (KUN).

**Table 1:** Samples used for phylogenetic analyses of the *A. sikkimense* complex and the data generated from them.

Taxa	Individual codes	Voucher ID	Collecting site	Altitude (m)	Genome size/Gb	Ploidy level	Available data			Sample name in GSA (submission)
							nrDNA	pt genome	Transcriptome	
***A. sikkimense***	HY1	MXG22-32	Hongyuan, Sichuan	3,858	11.08	(4×)	✓	✓	✓	SIKHY1
	HY2	MXG22-32	Hongyuan, Sichuan	3,858	10.99	(4×)	✓	✓	✓	SIKHY2
	T1_JL1	MXG22-49	Jiulong, Sichuan	4,480	7.35	(2×)	✓	✓	✓	SKIJC1
	T1_JL2	MXG22-49	Jiulong, Sichuan	4,480	7.35	(2×)	✓	✓	✓	SKIJC2
	T1_JL3	MXG22-49	Jiulong, Sichuan	4,480	–	–	✓	✓	✓	SKIJC3
	T1_JL4	MXG22-49	Jiulong, Sichuan	4,480	–	–	✓	✓	✓	SKIJC4
	T2_JL1	MXG22-51	Jiulong, Sichuan	4,335	16.3	(4×/6×)	✓	✓	✓	BEEJC1
	T2_JL2	MXG22-51	Jiulong, Sichuan	4,335	–	–	✓	✓	–	BEEJC2
	T2_JL3	MXG22-51	Jiulong, Sichuan	4,335	16.11	(4×/6×)	✓	✓	✓	BEEJC3
	T2_JL4	MXG22-45	Kangding, Sichuan	3,819	16.21	(4×/6×)	✓	✓	✓	SKIXB1
	T1_YJ1	MXG22-56	Yajiang-Litang, Sichuan	4,185	8.03	(2×)	✓	✓	✓	SKIYJ1
	T1_YJ2	MXG22-56	Yajiang-Litang, Sichuan	4,185	7.42	(2×)	✓	✓	✓	SKIYJ2
	T1_YJ5	MXG22-56	Yajiang-Litang, Sichuan	4,185	–	–	✓	✓	✓	SKIYJ5
	T2_YJ3	MXG22-58	Yajiang-Litang, Sichuan	4,185	12.69	(4×)	✓	✓	✓	SKIYJ3
	T2_YJ4	MXG22-58	Yajiang-Litang, Sichuan	4,185	14.62	(4×)	✓	✓	✓	SKIYJ4
	BM1	MXG22-41	Daofu, Sichuan	3,891	6.47	(2×)	–	✓	✓	SKIBM1
	BM2	MXG22-41	Daofu, Sichuan	3,891	6.59	(2×)	✓	✓	✓	SKIBM2
	DC1	MXG22-59	Daocheng, Sichuan	4,471	14.57	(4×)	✓	✓	✓	SKIDC1
	DC2	MXG22-59	Daocheng, Sichuan	4,471	–	–	✓	✓	✓	SKIDC2
	BW1	MXG22-61	Daocheng, Sichuan	4,337	–	–	✓	✓	✓	SKIBW1
	BW2	MXG22-61	Daocheng, Sichuan	4,337	14.43	(4×)	✓	✓	✓	SKIBW2
	TG	MXG22-43	Kangding, Sichuan	3,704	13.6	(4×)	✓	✓	✓	SKITG1
***A. beesianum***	JZS	MXG-2209-15	Kunming, Yunnan	4,300	–	4×	✓	✓	–	BEJZS1
***A. yuanum***	HY1	MXG22-33	Hongyuan, Sichuan	3,855	6.37	(2×)	✓	✓	✓	YUAHY1
	HY2	MXG22-33	Hongyuan, Sichuan	3,855	6.39	(2×)	✓	✓	✓	YUAHY2
***A. jichouense***	JC1	MXG22-46	Jiulong, Sichuan	4,490	21.38	8×	✓	✓	✓	JICJC1
	JC2	MXG22-46	Jiulong, Sichuan	4,490	–	–	✓	✓	✓	JICJC2
	JC3	MXG22-46	Jiulong, Sichuan	4,490	21.45	8×	✓	✓	✓	JICJC3
***A. zhegushanense***	ZG1	MXG22-27	Maerkang, Sichuan	3,192	8.01	2×	✓	✓	✓	ZHEZG1
	ZG2	MXG22-27	Maerkang, Sichuan	3,192	–	–	✓	✓	✓	ZHEZG2
	HY1	MXG22-35	Hongyuan, Sichuan	3,271	–	–	✓	✓	✓	ZHEHY1
	HY2	MXG22-35	Hongyuan, Sichuan	3,271	7.99	2×	✓	✓	✓	ZHEHY2
***A.* cf*. aciphyllum***	TG	MXG22-42	Kangding, Sichuan	3,694	13.3	(4×)	–	✓	✓	NOVTG1
	KD	MXG22-44	Kangding, Sichuan	3,395	10.02	(3×)	✓	✓	✓	NOVKD1
	HM	MXG22-56	Yajiang, Sichuan	4,266	12.49	(4×)	✓	✓	✓	HM2201
***A.* cf*. henryi***	GW1	MXG22-23	Nanjiang, Sichuan	2,283	8.85	2×	✓	✓	✓	PLUGW1
	GW2	MXG22-23	Nanjiang, Sichuan	2,283	8.9	2×	✓	✓	✓	PLUGW2
***A.* sp.** nov.	YD	MXG22-62	Daocheng, Sichuan	3,396	14.46	4×	✓	✓	✓	CYAYD1
*A. stenodon*	WTS	H14-0803	Yuxian, Hebei	1,930	–	–	✓	✓	–	A1
*A. cyaneum*	YZ	H18-0723	Yuzhong, Gansu	2,410	–	–	✓	✓	–	A2
*A. forrestii*	JC	MXG22-52	Jiulong, Sichuan	4,335	–	–	✓	✓	✓	FORJC1
	YL	no voucher	Yulong, Yunnan	3,372	–	–	–	✓	✓	YLWZ1

Ploidy level in brackets are estimated from flow cytometric analyses. Short lines (-) denote unacquired data.

**Figure 2:**
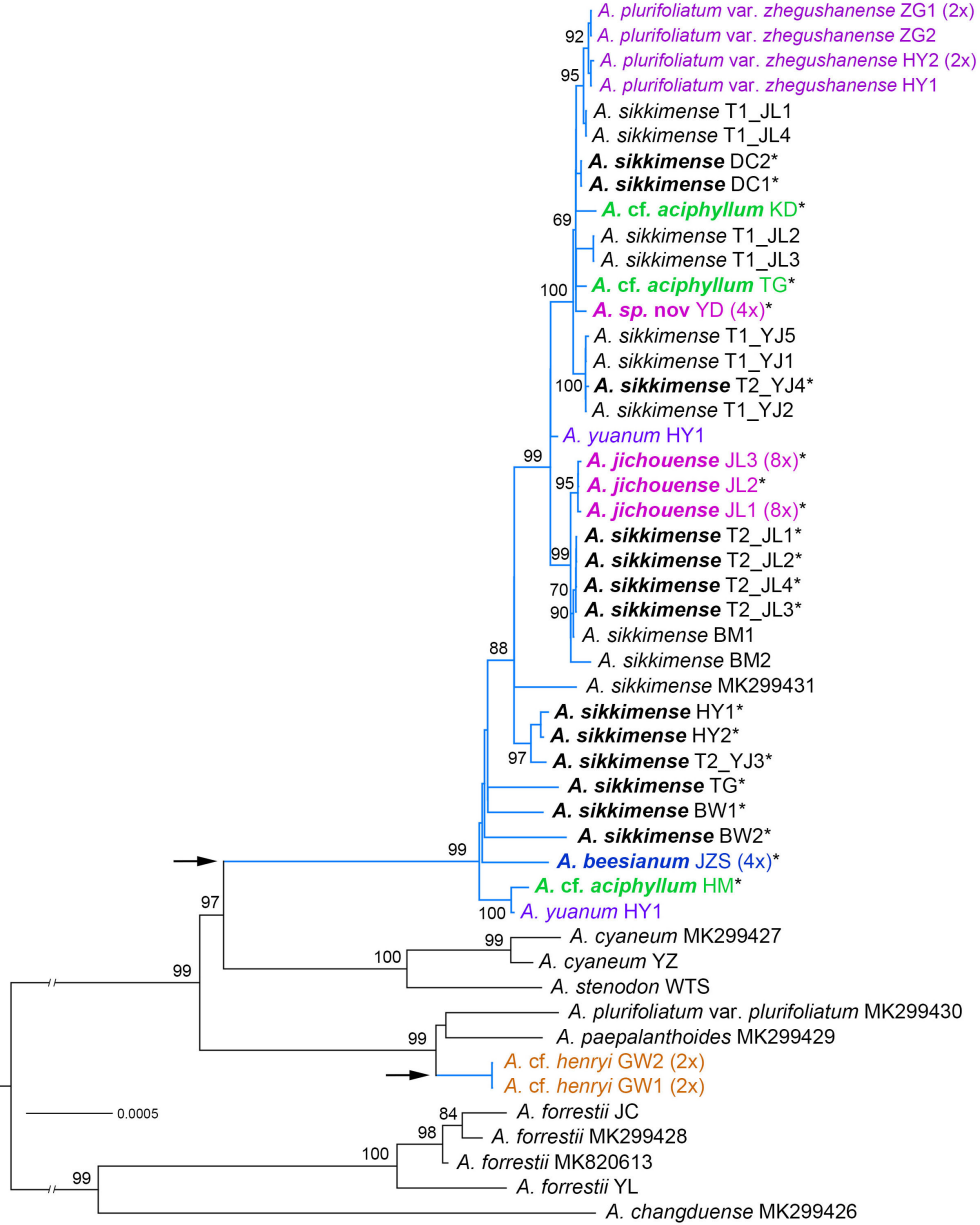
Phylogenetic relationships of the *Allium* sect. *Sikkimensia* inferred by maximum likelihood (ML) based on plastome CDS sequences. The *A. sikkimense* complex is represented by baby blue branches (also shown by the arrows), with scientific names in different colors indicating non–*A. sikkimense* species. Scientific names given in bold italics indicate potential polyploid taxa (also shown by the asterisks in their upper right corner). Ploidy levels (2× to 8×) in brackets are indicated next to the taxa names. T1/T2 after scientific names indicate different morphotypes of a sympatric population. Support values of the main nodes are shown along the branches. The seven species with GenBank accession numbers are downloaded from NCBI.

To test the phylogenetic hypothesis of sect. *Sikkimensia* proposed by Xie et al. (2019) and assess the monophyly of the *A. sikkimense* complex, we carry out a phylogenetic reconstruction of sect. *Sikkimensia* using nrDNA and plastome data, respectively, with *A. forrestii* and *A. changduense* selected as outgroup taxa according to Huang et al. (2022). A total of 106 nuclear ribosomal internal transcribed spacer (ITS) and 108 external transcribed spacer (ETS) sequences (39 newly sequenced, respectively; the rest obtained from the GenBank database) (**Tables 1**, **Supplementary Table S2**), and 49 plastomes (42 newly assembled; seven taken from the GenBank as shown in **Figure 2** with the National Center for Biotechnology Information (NCBI) accession numbers) are included in the present study to represent all recognized species of sect. *Sikkimensia*. Then, 36 transcriptomes from seven species (apart from *A. beesianum* not successfully sequenced) of the *A. sikkimense* complex, two transcriptomes of *A. forrestii* as outgroups are used to reconstruct its genome-scale phylogeny, and 28 samples from seven species (except *A. beesianum*) are used for genome size analysis. Detailed information on sampling is given in **Table 1**.

### 2.2 Whole-genome resequencing and transcriptome sequencing

The same quick-frozen samples were used for total DNA and RNA extraction, respectively. The additional three samples for whole-genome resequencing were extracted from silica gel–dried leaves. For whole-genome resequencing, total DNA was sheared into fragments using the Covaris system. The sheared DNA fragments were used to construct short-insert libraries (350 bp) and sequenced using Illumina NovaSeq 6000 (Illumina, San Diego, CA, USA). The resequencing work was carried out by Novogene (Tianjin, China). A data size of 2 Gb was sequenced for each sample. Whole-genome resequencing data from the 42 samples were deposited in the Genome Sequence Archive (Chen et al., 2021b) in National Genomics Data Center (NGDC) (CNCB-NGDC Members and Partners, 2022), China National Center for Bioinformation/Beijing Institute of Genomics, Chinese Academy of Sciences (GSA: CRA013414) that are publicly accessible at https://ngdc.cncb.ac.cn/gsa.

For transcriptome sequencing, total RNA was extracted using TRIzol reagent (Invitrogen, CA, USA) following the manufacturer’s procedure. After quality evaluation, the cDNA library was prepared using the Illumina TruSeq™ RNA Sample Preparation Kit (Illumina, San Diego, CA, USA). The transcriptomes were sequenced using Illumina NovaSeq 6000 (Illumina, San Diego, CA, USA) platforms at Novogene (Tianjin, China). All samples were sequenced to a data size of 8 Gb (150-bp paired-end reads). Transcriptome sequencing from the 38 samples were deposited at the NGDC under accession number CRA013238.

### 2.3 Plastome and nrDNA assembly, and phylogenetic analyses

For whole-genome resequencing data, the adapter sequences and paired reads containing more than 10% N and/or with a proportion of low quality (Phred quality <5) bases greater than 50% were removed using fastp 0.23.2 (Chen et al., 2018). The plastomes and nuclear ribosomal RNA (ETS, 18S, ITS1, 5.8S, ITS2, and 26S) sequences were assembled from the clean data using GetOrganelle (Jin et al., 2020). We used PGA (Plastid Genome Annotator) (Qu et al., 2019) to annotate the plastomes and used a previously published plastome of *A. sikkimense* (NC_058221.1) as a reference. The coding sequences (CDSs) were extracted from the GenBank file using a custom script. The single-CDS matrix of all the samples was aligned using MAFFT 7.505 (Katoh and Standley, 2013) and trimmed using TrimAI v1.4.1 (Capella-Gutiérrez et al., 2009). Geneious R8 (Kearse et al., 2012) was used to generate the final dataset. A maximum likelihood (ML) phylogenetic tree was constructed using RAxML v.8.2.12 (Stamatakis, 2014) with a general time-reversible model with gamma-distributed rate variation (GTRGAMMA), and node supports were calculated from 1,000 bootstrap replicates. Phylogenetic analyses of nrDNA were estimated using maximum parsimony (MP) and Bayesian inference (BI) with a best-model of HKY+I+G following the procedure of Huang et al. (2022).

### 2.4 Homology inference and phylotranscriptomics analyses

We followed the “phylogenomic dataset construction” pipeline (Yang and Smith, 2014) for transcriptome data filtering, *de novo* assembly, translation, and homology and orthology inference. The pipeline can be found on the website https://bitbucket.org/yanglab/phylogenomic_dataset_construction/src/master/.

Read processing in this pipeline was used to correct sequencing errors (Rcorrector, Song and Florea, 2015), trim sequencing adapters and low-quality sequences (trimmomatic v 0.39, Bolger et al., 2014), filter organelle reads (Bowtie2 v 2.3.5.1, Langmead et al., 2019), check the read quality (fastqc v 0.11.6, https://www.bioinformatics.babraham.ac.uk/projects/fastqc/), and remove over-represented reads. After read processing, *de novo* assembly of each sample was preformed using Trinity v 2.5.1 (Grabherr et al., 2011). After quality analysis with Transrate v1.0.3 (Smith-Unna et al., 2016), the poorly supported and chimeric transcripts were removed (Yang and Smith, 2013). Filtered transcripts were clustered using Corset v 1.09 (Davidson and Oshlack, 2014) and a representative transcript with the longest length was extracted for each putative gene. Salmon v 0.9.1 (Patro et al., 2017) was also used in this step to remap filtered reads to filtered transcripts. Transcripts were translated using Transdecoder 5.3.0 (https://github.com/TransDecoder/TransDecoder) and a custom blast database generated from garlic (*Allium sativum*) (Sun et al., 2020). Only the open reading frames with blast hits were retained for homology inference. The CD-HIT v 4.6.8 (Fu et al., 2012) was used to reduce redundancy, and homology inference was preformed using an all-by-all BLASTN search for CDSs. Putative homolog groups were then clustered using MCL v1.37 (Van Dongen, 2000). Clusters were aligned with MAFFT v 7.508 (Katoh and Standley, 2013) and construct tree using RAxML v.8.0.17 (Stamatakis, 2014). Tips were trimmed using relative and absolute length cutoffs. Orthologs were inferred using the 1to1 method (Yang and Smith, 2014). The minimum number of taxa in each orthology alignment was set to 34 (>0.85% of all the samples).

A concatenated matrix was generated from all the cleaned orthology alignments. ML tree construction was performed using RAxML v.8.0.17. The GTRGAMMA model was used for sequence evolution, and node supports were calculated from 100 bootstrap replicates. The ML tree was also reconstructed for each cleaned orthology alignment using RAxML. Gene trees were then used to infer a coalescent species tree using ASTRAL-III v.5.7.1 (Zhang et al., 2018). Local posterior probabilities (Sayyari and Mirarab, 2016) were used to assess clade support.

### 2.5 Genome size measure and chromosome counts

Genome sizes of selected samples were measured using the BD FACSCalibur flow cytometer (Franklin Lakes, USA). The protocol was the same as in our previous study (Huang et al., 2022).

Living plants were collected and subsequently cultivated indoors to obtain the fresh root tip cells, which were pretreated with a saturated solution of paradichlorobenzene for approximately 9 h at 4°C in a refrigerator, and fixed with a mixture of absolute ethyl alcohol and glacial acetic acid (3:1, v/v) for 15 h at room temperature. The samples were then hydrolyzed in HCl of 1 mol/L at 60°C for 9 min, stained with modified Carbol fuchsin solution for about approximately 2 h, and then squashed with a drop of 45% acetic acid. At least five metaphase plates from each individual were examined for chromosome number counting.

### 2.6 Phylonet analyses

We used the software PhyloNet v 3.6.9 (Than et al., 2008) to reconstruct phylogenetic networks in the presence of reticulate evolutionary events. PhyloNet can also take account for ILS in the analyses. Because of computational limitations, we selected 20 samples that represent all the species or clades in this analysis. We re-preformed the procedure of Yang and Smith (2014) for these samples. The minimum number of taxa was set to 20 (no missing), and the minimum cleaned alignment length was 500 bp for all the orthology alignments.

We reconstructed the ML phylogenetic gene trees for each orthology alignment using IQ-Tree v 1.6.1 (Nguyen et al., 2015). These trees were used as input to Phylonet. The species networks, with the optimal number of hybridization events 0–5, were inferred using a maximum pseudo-likelihood (MPL) method (Yu and Nakhleh, 2015). We used the “CalGTProb” command in PhyloNet to calculate the probability scores for each network (Yu et al., 2012). The best-fit species network model was evaluated using the Akaike information criterion (Akaike, 1998).

### 2.7 QulBL analysis

We used the Quantifying Introgression via Branch Lengths approach to distinguish the models with ILS + introgression from those with ILS alone (Edelman et al., 2019). Nine samples representing all species or clades were used in this analysis. The orthology alignments of these nine samples were generated from the datasets used in the phylonet analyses. The ML trees for each orthology alignment were also reconstructed using IQ-Tree v 1.6.1 (Nguyen et al., 2015) and used as the input tree file in the QulBL analysis. The ILS + introgression model was accepted as a better explanation for the data if the Bayesian information criterion (BIC) test with a strict cutoff of ΔBIC < −10. Otherwise, the gene topologies that did not fit the species tree were mainly caused by ILS.

## 3 Results

### 3.1 Chromosome counts, genome sizes, and ploidy levels

The chromosome numbers of several species of sect. *Sikkimensia* are counted as diploid (2n = 2x = 16) for *A. plurifoliatum* var. *zhegushanense* (Hongyuan and Maerkang, Sichuan) (**Supplementary Figures S1A, B**) and *A.* cf. *henryi* (Nanjiang, Sichuan) (**Supplementary Figure S1C**); tetraploid (2n = 4x = 32) for *A.* sp. nov. (Daocheng, Sichuan) (**Supplementary Figure S1D**) and *A. beesianum* (Kunming, Yunnan) (**Supplementary Figure S1E**); and octoploid (2n = 8x = 64) for *A. jichouense* (Jiulong, Sichuan) (**Supplementary Figure S1F**).

The flow cytometric analyses show that the genome size of octoploid *A. jichouense* is the largest (21.38–21.45 Gb), whereas the mean genome size of two diploid species is 8.44 Gb, ranging from 7.99–8.01 Gb (*A. plurifoliatum* var. *zhegushanense*) to 8.85–8.9 Gb (*A.* cf. *henryi*). The tetraploid *A.* sp. nov. has 1.71 times the genome size of the diploid species, and the octoploid *A. jichouense* has 2.54 times the genome size, suggesting a markedly positive linear correlation trend (*R* ^2 = ^0.9943) between genome size and ploidy level among the species studied within sect. *Sikkimensia*. Thus, we conclude that the genome sizes of those chromosome numbers unknown samples as more than one fold high as that of diploid species can probably all be polyploid. Genome sizes and estimates of DNA ploidy levels according to flow cytometric analyses are shown in **Table 1**.

### 3.2 Phylogenetic inferences of sect. *Sikkimensia* based on plastome and nrDNA

**Figure 3:**
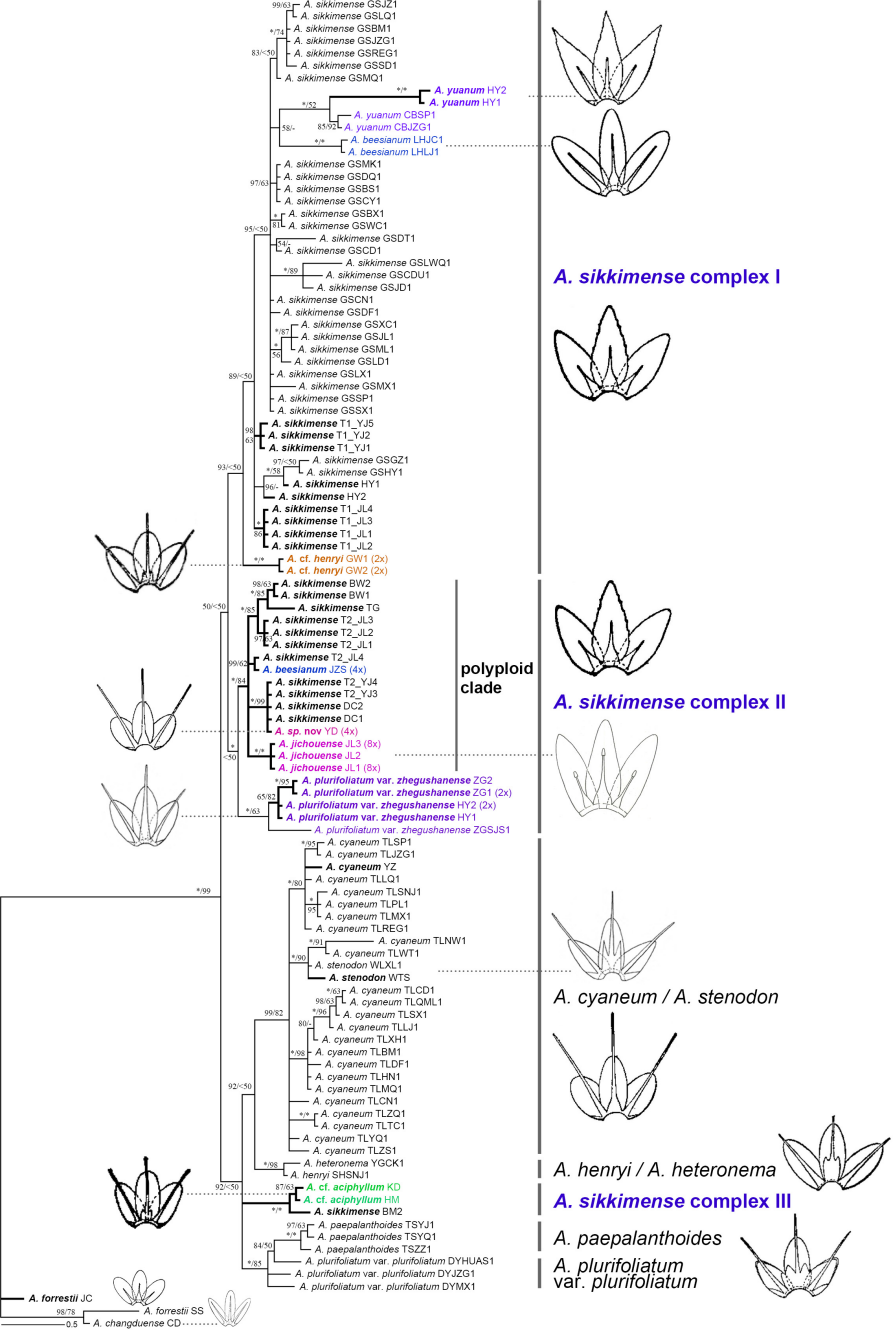
Phylogenetic relationships of the *Allium* sect. *Sikkimensia* inferred from Bayesian analysis based on nrDNA sequences. *Allium sikkimense* complex I–III represents three identified genetic clusters. Black bold branches and scientific names given in bold italics indicate the samples obtained in this study, whereas scientific names in different colors indicate non–*A. sikkimense* species of the *A. sikkimense* complex. Illustrations [modified from *FRPS* (Xu, 1980) and *FOC* (Xu and Kamelin, 2000)] indicate the different types of filaments (included or exserted) of major lineages or taxonomic units. Ploidy levels (2× to 8×) in brackets are indicated next to the taxa names. T1/T2 after scientific names indicate different morphotypes of a sympatric population. Bayesian posterior probabilities (PP) and bootstrap values (BS) values are shown along the branches, respectively. Asterisks and short lines denote the values of 100 % for PP/BS and no support value, respectively.

The aligned plastome dataset used for phylogenetic tree reconstruction was 71,869 bp in length and consisted of 83 CDSs. The ML phylogenetic tree of the CDSs was shown in **Figure 2**. For the nrDNA dataset, the combined ITS + ETS alignment was 1,146 bp in length, and a total of 333 variable sites were identified, of which 208 (18.15%) were parsimony‐informative. Phylogenetic analysis of the nrDNA based on BI and MP revealed the overall same tree topology within sect. *Sikkimensia* (**Figure 3**).

Our plastome and nrDNA phylogenies support the recognition of *A. cyaneum* (including *A. stenodon*, the same below), *A. henryi*–*A. heteronema*, and *A. plurifoliatum* var. *plurifoliatum*–*A. paepalanthoides* as separate clades, respectively, whereas the *A. sikkimense* complex is shown to be non-monophyletic, particularly due to cyto-nuclear discordance regarding the placement of *A.* cf. *henryi* and *A.* cf. *aciphyllum* (see below).

In the plastome phylogeny (**Figure 2**), all the samples of the *A. sikkimense* complex form a large well-supported core clade, and are sister to the *A. cyaneum* clade, with the exception of *A.* cf. *henryi*, which is inferred to be sister to a clade consisting of *A. plurifoliatum* var. *plurifoliatum* and *A. paepalanthoides*. Within the complex core clade, species-level phylogenetic relationships are poorly resolved with many short internal branches and little substructure. Some morphospecies do not form species-specific lineages, particularly *A. sikkimense*, which is mutually embedded with other members, resulting in no clear species relationships and thus no further phylogenetic signal on genome size variation (corresponding to ploidy level). Nevertheless, three relatively supported subclades are also revealed, namely the *A. sikkimense* partial samples (T2_JL) from Jichou Mt. of Jiulong, *A. jichouense*, and *A*. *plurifoliatum* var. *zhegushanense*.

Compared to the plastome phylogeny, the nrDNA-based phylogeny shows a relatively clear phylogenetic divergence, although the *A. sikkimense* complex is also non-monophyletic (**Figure 3**). Almost all morphospecies form taxon-specific lineages except *A. sikkimense*, which is placed in three different genetic clusters, as is the *A. sikkimense* complex (I–III). *Allium sikkimense* complex I (including *A. sikkimense*, *A. beesianum*, *A. yuanum*, and *A.* cf. *henryi*) forms a sister to *A. sikkimense* complex II, which contains a diploid *A. plurifoliatum* var. *zhegushanense* clade and a well-supported polyploid clade (including five polyploid populations of *A. sikkimense*, *A. beesianum*-JZS, *A. jichouense*, and *A.* sp. nov.), whereas *A. sikkimense* complex III (including *A.* cf. *aciphyllum* and *A. sikkimense*-BM) forms a polytomy with two other major clades, *A. cyaneum* + *A. henryi*–*A. heteronema* and *A. plurifoliatum* var. *plurifoliatum*–*A. paepalanthoides*, but both with only weak support values.

There are substantial differences (i.e., gene tree conflicts) between the plastome and nrDNA phylogenies; however, in both phylogenies, the partial samples of *A. sikkimense* from Jichou Mt. (Morphotype 2, i.e., T2_JL) and *A. jichouense* from Jichou Mt. of Jiulong are always placed in the same clade with strong support values, although they do not form reciprocally monophyletic sister clades, whereas the *A*. *plurifoliatum* var. *zhegushanense* samples from two different populations always stably form their own clade.

### 3.3 Phylogenomic inferences of the *A. sikkimense* complex based on transcriptome data

**Figure 4:**
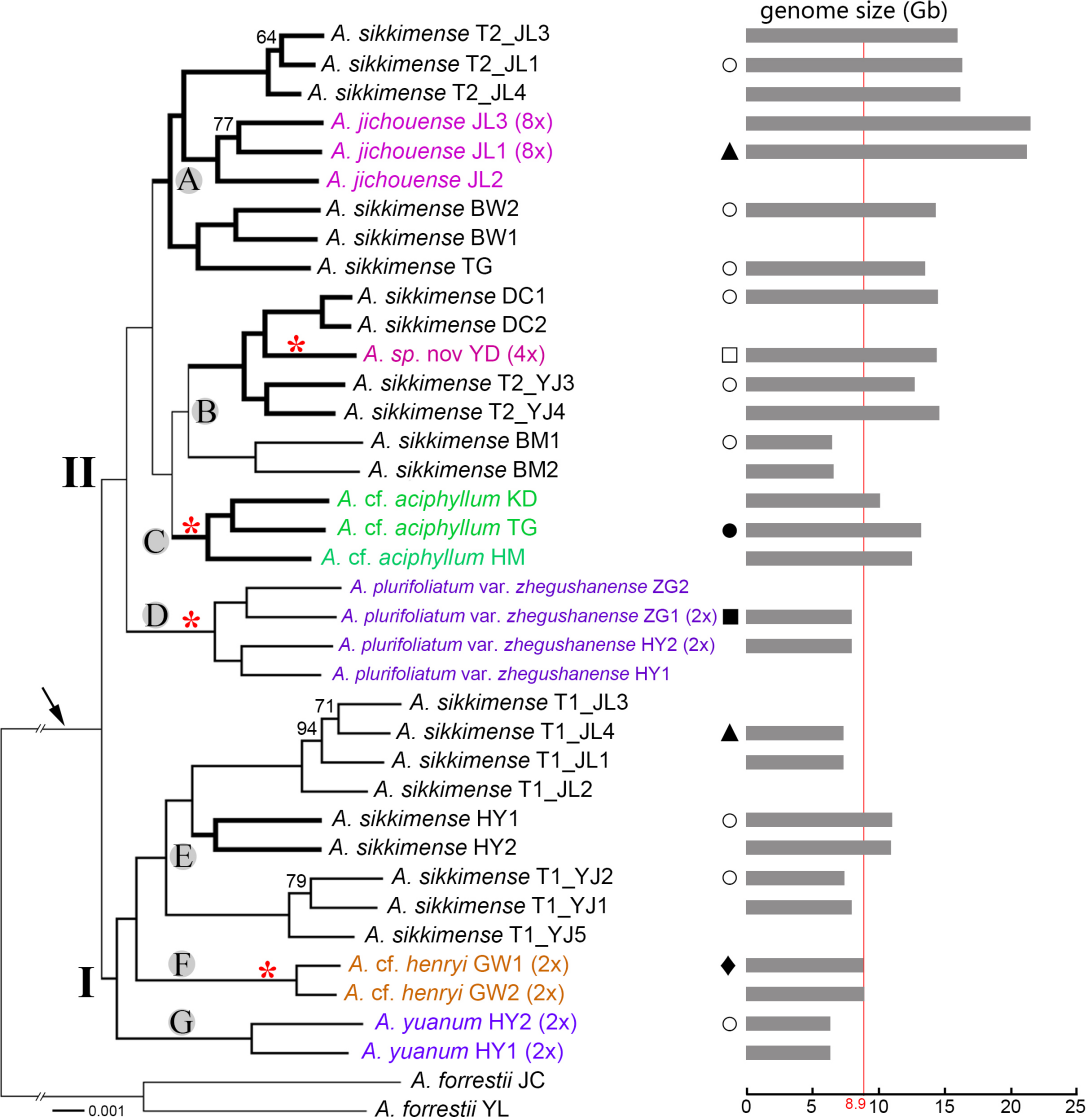
Transcriptome phylogenetic relationships of the *A. sikkimense* complex (shown by the arrow). Genome sizes and habitat types are mapped on the phylogenetic tree. Two major clades (I and II) and seven subclades **(A–G)** represent the identified genetic clusters. Bootstrap percentages (BPs) are given along the branches, and only values below 95 BP are shown. Scientific names given in different colors indicate non–*A. sikkimense* species. Potential polyploid branches are represented by a thicker line. Ploidy levels (2× to 8×) in brackets are indicated next to the taxa names. Genome sizes (1C, Gb) are shown as grey bars on the right side with the maximum diploid genome size indicated by red line. Red asterisks along branches indicate the taxa with exserted filaments. T1/T2 after scientific names indicate different morphotypes of a sympatric population. Graphical symbols beside the tree represent habitat types as follows: hollow circles (○), moist slope in scrub and meadows; solid triangles (▴), alpine screes; hollow square (□), arid slope under pine forests; solid square (▪), shady and moist slope under forests; solid circle (•), arid rock slope along river valley; solid diamond (♦), sunny rock slope.

After filtering for alignment length (>300 bp) and minimum number of taxa (34), we obtained 1,087 orthologs for the final phylogenomic inference. The concatenated alignment for ML tree construction was 838,585 in length. The ML-derived transcriptome tree (**Figure 4**) was topologically similar to the ASTRAL species tree (**Supplementary Figure S2**), differing only in the placement of *A. yuanum* and *A.* cf. *henryi*. The phylogenetic relationships within the *A. sikkimense* complex revealed by the transcriptome data are better resolved than those derived from the plastome data and the nrDNA-based phylogeny. It is generally similar to the result of the nrDNA-based phylogeny in delimiting two major clades in the *A. sikkimense* complex, differing from the latter mainly in the placement of the *A.* cf. *aciphyllum*–*A. sikkimense* (BM2) clade (**Figure 3**).

Within the *A. sikkimense* complex, two well-resolved clades is clear, and seven well-diverged subclades are identified (**Figure 4**). Subclade A, the polyploid-only *A. sikkimense*–*A. jichouense* branch, consists of two polyploid *A. sikkimense* lineages and one octoploid *A. jichouense* lineage. Subclade B contains the polyploid *A. sikkimense*–*A.* sp. nov. branch and a diploid *A. sikkimense* lineage. Subclades C-G each contain a single species, corresponding to the *A.* cf. *aciphyllum* (polyploid), *A. plurifoliatum* var. *zhegushanense* (diploid), *A. sikkimense* (diploid-polyploid), *A.* cf. *henryi* (diploid), and *A. yuanum* (diploid) lineages, respectively.

In general, all morphospecies form taxon-specific lineages with maximum support, with the exception of *A. sikkimense*, which is placed in three different subclades A, B, and E. The different morphotypes of *A. sikkimense* in the same location show markedly genetic differentiation. For instance, the five sampled individuals of *A. sikkimense* from Yajiang (YJ) have clearly different leaf shapes and genome sizes, three (Morphotype 1, i.e., T1_YJ1-2, 5) of which with broad leaves and small genome sizes (diploid) are placed in the subclade E of clade I (**Figures 1**, **4**), whereas the other two (Morphotype 2, i.e., T2_YJ3-4) with narrow leaves and large genome sizes (polyploid) are placed in the subclade B of clade II (**Figures 1**, **4**), which is also identical with the result of the nrDNA-based phylogeny. Furthermore, two morphotypes of *A. sikkimense* from Jichou Mt. of Jiulong has distinctly different characters (e.g., body size, flower size and color), habitat preference (shrub vs. alpine screes), and genome sizes. Four sampled individuals (Morphotype 1, i.e., T1_JL1-4), which grow on alpine screes and have small flowers and genome sizes (diploid), are placed in the subclade E of clade I, whereas the other three individuals (Morphotype 2, i.e., T2_JL1, 3-4), which grow under shrub and have large flowers and genome sizes (4×/6×), are placed in the subclade A of clade II.

### 3.4 Network evolution analysis

**Figure 5:**
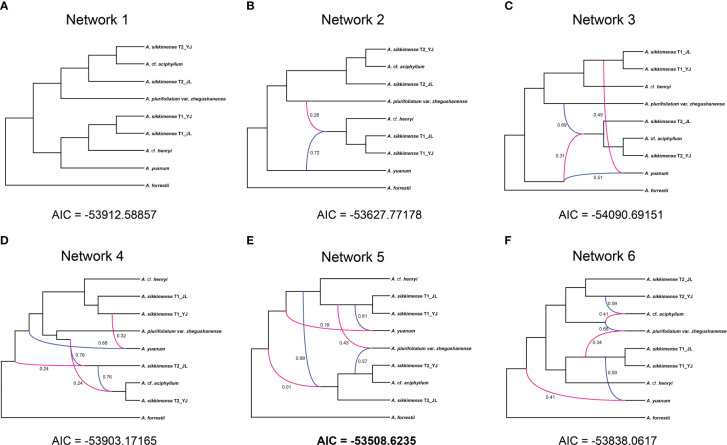
Inheritance probabilities inferred based on Phylonet analyses with the reticulations represented by lines. **(A)** With no reticulation. **(B–F)** One to five reticulation events allowed.

All networks with reticulation events were shown to be a better scenario than a strictly bifurcating tree in our phylonet analyses (**Supplementary Table S3**). The best fitting network was found when the maximum number of four reticulations was added and it had three reticulation events (**Figure 5E**). The reticulation event was found in diploid species or the ancestral species in several different networks (**Figure 5**).

### 3.5 QulBL analysis

Using 824 gene trees as input, QulBL analysis revealed that none of the triplets tested showed significant evidence of post-speciation introgression (ΔBIC all greater than −10) in selected samples (**Supplementary Table S4**).

## 4 Discussion

### 4.1 *Allium sikkimense* contains more than one species

Xie et al. (2019) found that *A. beesianum*, *A. yuanum*, *A. plurifoliatum* var. *zhegushanense*, and *A. sikkimense* formed a polytomy in the chlorotype tree. The phylogenetic relationships of these species were also poorly resolved in their ribotype (nrDNA) tree. Huang et al. (2022) proposed a new species *A. jichouense* and found that this new species was also embedded in different populations of *A. sikkimense* based on a few of gene fragments. Through extensive sampling of different species in the Hengduan Mountains, our transcriptome-based phylogenetic tree found that there are up to six species embedded in different populations of *A. sikkimense.* The use of more than 1,000 orthologous genes in the phylogenomic analyses excluded the possibility of a lack of informative sites in the dataset. On the contrary, our study revealed a highly supported relationship for these species using both concatenation and coalescent approaches (**Figures 4**, **Supplementary Figure S2**). Although we did not obtain the transcriptomic material of *A. beesianum*, this species may also be embedded in different populations of *A. sikkimense*, according to the result of Xie et al. (2019) and our phylogenetic analyses of plastome and nrDNA (**Figures 2**, **3**).

Three possible explanations may be accounted for the inclusion of the other seven species in different populations of *A. sikkimense*. The first possibility is incomplete lineage sorting. ILS can lead to poorly resolved phylogenies (polytomy) in rapidly radiating groups (e.g., Whitfield and Lockhart, 2007; Gagnon et al., 2022). However, ILS is a stochastic process and rarely leads to clear species delimitation and unambiguously resolved relationships for multiple embedded species with high support, especially considering that these embedded species each formed a separate branch (**Figure 4**). The second possibility is that *A. sikkimense* has hybridized with seven other species. All the embedded species formed a separate branch rather than intermingling. The embedded species are all good morphospecies and no morphologically intermediate individuals were found between the embedded species and *A. sikkimense*. The QulBL analysis also failed to detect any recent hybridization signal for these species. Taken together, embeddedness of these species within *A. sikkimense* is unlikely to be the result of recent introgression among these species. The embedded species did not form any sister species relationships in the phylogenetic trees, so the embeddedness is unlikely to be caused by ancient hybridization events between the ancestors of the embedded species and *A. sikkimense*. Furthermore, some embedded species showed no geographic contact with their respective related *A. sikkimense* samples. For example, some *A. sikkimense* samples collected from the Hengduan Mountains showed the closest relationship to *A.* cf. *henryi*, which is collected from central China. An allopatric divergence scenario is more acceptable for their sister relationship rather than for hybridization. Overall, we point out that it is virtually impossible for *A. sikkimense* to hybrid with all seven species, although partial embeddedness could be caused by hybridization.

The most reasonable possibility is that *A. sikkimense per se* is actually a species complex containing more than one species. We point out that there are at least two cryptic species existed within *A. sikkimense*. The samples of *A. sikkimense* fall into two major clades of the *A. sikkimense* complex (**Figure 4**). These two clades all contain diploid samples of *A. sikkimense* and may have different origins. In clade I, all the samples of *A. sikkimense* form a clade and this clade should represent a cryptic species in morphologically recognized *A. sikkimense*. The *A. sikkimense* samples did not form a monophyletic branch in clade II. A very complex relationship and change in ploidy level (or genome size) was found for *A. sikkimense* and embedded species in clade II. Some samples of *A. sikkimense* were found to be sister to *A.* cf. *aciphyllum* (subclades B–C in **Figure 4**), a species with completely different morphological characteristics (e.g., conspicuously exserted filaments). *Allium* sp. nov., another species with exserted filaments, was also embedded in this branch. The other *A. sikkimense* samples formed a subclade with the *A. jichouense* samples (subclade A in **Figure 4**). We cannot be sure, based on current data, whether the *A. sikkimense* samples in clade II are the same species or different species. *Allium sikkimense* samples in clade II are very closely related based on a relatively shorter branch length than clade I and their very closely related relationships in the plastome tree. However, it is really very difficult to explain the inclusion of three other species in this clade. We point out that very complex ploidy changes (including autopolyploidization and allopolyploidization) may have already been involved in the evolution of this clade.

### 4.2 Polyploidy, possible budding speciation, and habitat differentiation in *Allium sikkimense* complex

Genome size is related to the expansion of transposable elements and whole-genome duplication (Grover and Wendel, 2010). Limited variation was found for the genome size of diploid species in the *A. sikkimense* complex (6–8 G), so the genome sizes with significant multiple increase are polyploidy. We found a rich diversity of polyploidy in the *A. sikkimense* complex, especially for clade II in our transcriptome-based phylogenetic tree.

Some subclades consisted only of polyploid samples in clade II, such as the subclade A in **Figure 4**, which included samples of *A. sikkimense* and *A. jichouense*, and the subclade B, which included *A. sikkimense* and *A.* sp. nov. from Daocheng. More interestingly, recurrent polyploidization (octaploidy) was found within clade II. Polyploidization can lead to cryptic species diversity that cannot be detected from morphological characters alone (e.g., Ma et al., 2015), but polyploidy can also lead to obvious morphological and physiological changes (Van de Peer et al., 2017), such as larger size and flower, and greater drought tolerance (Baker et al., 2017). *Allium jichouense* is an octoploid with very specific morphological characteristics and has shown many adaptive traits to alpine screes (Huang et al., 2022). This species occurs in a completely different habitat than its closest relative *A. sikkimense* T2_JL. The samples of *A. sikkimense* T2_JL in this study were collected from the same mountain slope with *A. jichouense* and grows among shrubs. As pointed out by Han et al. (2020), polyploidy is probably to promote the ecological niche differentiation in *Allium*. It may also occur within the *A. sikkimense* complex, allowing new polyploids to colonize novel habitats, such as alpine screes for *A. jichouense*. *Allium jichouense* may be a species that arose directly from polyploidy, resulting in immediate reproductive isolation and ecological change.

The speciation of *A. jichouense* may have involved another special speciation process: budding speciation (Mayr, 1954; Crawford, 2010), defined as a derivative species forms within or near the edge of the progenitor species (via ploidy shifts, phenological changes, ecological differentiation, and/or niche shifts) (Anacker and Strauss, 2014). The progenitor-derivative mode has often been inferred in pairs of closely related nested species with overlapping ranges (Otero et al., 2022). Huang et al. (2022) have discussed that the habitat-restricted *A. jichouense* may be a species derived from the widespread and eurytopic *A. sikkimense* through budding speciation. In this study, we further confirm that this species is most closely related to the sympatrically distributed *A. sikkimense* T2_JL, which also possesses a large flower like *A. jichouense.* This morphological type of *A. sikkimense* has a much wider distribution than *A. jichouense* and occupies several lineages in clade II, so *A. jichouense* might have originated from this type of *A. sikkimense* by budding speciation. A change in ploidy level could also result in abrupt reproductive isolation between *A. jichouense* and the symmetrically distributed *A. sikkimense* T2_JL. Another possible example of budding speciation is *A.* sp. nov. from Daocheng, which is a tetraploid with a very narrow distribution and is embedded within the tetraploid samples of *A. sikkimense* from the adjacent region. It grows in sheltered habitats under the pine forest and has a phenotype similar to *A. cyaneum* (e.g., exerted filaments). The obviously exserted filaments of this species may be associated with changes in the pollination environment under the pine forest. This species also flowers later (September to October) than *A. sikkimense* (July to September), suggesting possible reproductive isolation in flowering phenology. From the habitat changes of these two species, we point out that ecological isolation may play an important role in the species diversity of the *A. sikkimense* complex.

Ecological divergence has been found to be an important mechanism of diversity in southwest China (Liu et al., 2014; Gao et al., 2015; Cai et al., 2018; Zhao et al., 2021). Apart from *A. jichouense*, *A.* sp. nov., and their correspondingly symmetrically distributed relatives (*A. sikkimense*), *A.* cf. *aciphyllum* and *A. sikkimense* can also be found in the same geographical site. However, *Allium* cf. *aciphyllum* usually grows in arid habitats on bare rock with little soil, which is very different from the habitat of *A. sikkimense* (in moist scrub and meadows). The flowering season of *A.* cf. *aciphyllum* (late August to October) is also later than that of the sympatric *A. sikkimense*. This polyploid species is more closely associated with xeric microhabitats and thereby possesses aridity-adapted traits such as distinctly reticulated bulb tunics, hard semicylindrical leaves, and delayed flowering time (i.e., drought escape). Furthermore, the habitat differentiation of the *A. sikkimense* complex is also drastic at the diploid level. All early branching species in two major clades are all diploid species, and many of them grow on different habitats. For example, *A. plurifoliatum* var. *zhegushanense* has a sympatric distribution with *A. sikkimense*, but *A. plurifoliatum* var. *zhegushanense* grows in shady forests, which is quite different from the sympatric *A. sikkimense* (in scrub and meadows).

Although we observed an interesting polyploidization-related scenario, it remains unclear whether polyploidy represents a real adaptive advantage in the case of *A. sikkimense* complex, because some polyploid populations in the complex show no ecological divergence compared to diploid populations. In other words, diploid species in the *A. sikkimense* complex can also adapt to different environments through other evolutionary changes. For example, we found two different morphological types of *A. sikkimense* within the same community in Yajiang. These two types of *A. sikkimense* each formed a branch and belong to two major clades in phylogenomic analyses, and they have different ploidy levels. They actually belong to two different species according to our phylogenomic analyses. The diploid samples of *A. sikkimense* from Jichou Mountains of Jiulong can also occur in alpine screes and grow on the same slope as the octoploid *A. jichouense*, but they usually grow in the microhabitat with more soil under gravel. The diploid *A. yuanum* can also occur sympatrically in the same community with the polyploid *A. sikkimense.*

Nevertheless, our study shows that co-occurring species in the *A. sikkimense* complex usually have different ploidy levels, suggesting that polyploidy is a very important way of promoting reproductive isolation of sympatric *Allium* species in the complex. Although *A. sikkimense* with different ploidy levels usually have completely different morphological characteristics within a particular geographical location, we did not find any pattern of morphological variation throughout the whole Hengduan mountain range. *Allium* species also have strong adaptability and phenotypic plasticity (under stress). This makes it very difficult to distinguish different cryptic species by morphological characters. Cytotypic variation is widespread within many angiosperm species or species complexes (e.g., Halverson et al., 2008). Intraspecific variation in ploidy level in different cryptic species within *A. sikkimense* was also observed in this study. Polyploidy in *Allium* species can have multiple origins (e.g., Wu et al., 2010), as studies have shown that multiple origins are the rule for most polyploids (Soltis et al., 2003). This further complicates the assessment of the evolutionary significance of polyploidy in *A. sikkimense*.

### 4.3 Challenges of phylogenetic reconstruction in *Allium* sect. *Sikkimensia*

Cytonuclear discordance is very common in plant groups involved in adaptive radiation, extensive hybridization, and polyploidisation (e.g., Rieseberg and Soltis, 1991; Gao et al., 2015; Ma et al., 2022). In our study, the plastomes of the *A. sikkimense* complex have their own evolutionary history and failed to resolve the phylogenetic relationships of the different species in the *A. sikkimense* complex thoroughly. The phylogenetic tree based on the plastomes shows a completely different relationship for these species and did not reveal any genetic structure related to species relationships, nor did it correspond to any ploidy pattern. The ptDNA data can provide a higher support relationship for distant species in the *Allium* sect. *Sikkimensia* (Xie et al., 2019). *Allium cyaneum*, *A. plurifoliatum* var. *plurifoliatum*, and *A. paepalanthoides* all have rather distant phylogenetic relationships to the species in the *A. sikkimense* complex in the plastome tree. Although the close related relationship of the species in the *A. sikkimense* complex was further confirmed in the plastome phylogenetic tree (**Figure 2**), there is still some discordance with the previous nuclear DNA–based backbone. *Allium* cf. *henryi* formed a clade with two other species (*A. plurifoliatum* var. *plurifoliatum* and *A. paepalanthoides*) distributed in central China. Plastid capture may have occurred between these species due to their close biogeographic relationships. The sequence differences of the plastomes among species in the core clade of the *A. sikkimense* complex are very small, leading to short internal branches and thus increasing topological conflicts, and the effect of ILS may be very strong in the ptDNA divergence of this adaptive radiation group.

Resolving the phylogenetic relationships of polyploid-rich plant lineages is very challenging even with high-throughput sequencing, especially for rapidly radiating groups (Španiel et al., 2023). The *A. sikkimense* complex may be a typical example of the evolutionary complexity of polyploid-rich plant lineages. Our study found no significant post-speciation gene flow between these species. ILS may be the main cause of gene tree discordance between orthologs. However, the phylogenetic relationship that allows for reticulation events always has a greater fitness to the dataset than a simple bifurcation in our Phylonet analyses. Reticulation events were mainly found between ancestral species, which cannot be detected by QulBL analysis. We also found that the species or ancestral species involved in reticulation events are unstable when different numbers of reticulation events are allowed in Phylonet analyses, suggesting that a very complex evolutionary history has taken place in this group. In addition, certainly there should be some uncollected samples and extinct species or lineages involved in these reticulation events.

Overall, our study revealed a well-resolved phylogenetic framework for species in the *A. sikkimense* complex, and we confirmed that polyploidization has played a crucial role in the diversification of this complex. Meanwhile, ecological isolation and possible budding speciation may also play a role in the rapid diversification of this complex. Our results also show that, in sect. *Sikkimensia*, the included or exerted filaments having long been used as an important diagnostic characters at the species level, are extremely unreliable taxonomically due to their extensive convergent and parallel evolution and are likely to have evolved multiple times independently within the section and should therefore be used with caution in the species delimitation. More extensive sampling and further in-depth studies are needed to confirm the taxonomic identity of some species such as *A.* sp. nov. and *A.* cf. *henryi*, which represent two potential new species based on both molecular data and morphological characters. On the basis of our study, we point out that *A. plurifoliatum* var. *zhegushanense* should not be treated as a variety of *A. plurifoliatum* and should be elevated to species rank.

### 4.4 Taxonomic treatment

***Allium zhegushanense* (J. M. Xu) D.Q. Huang & X.G. Ma**. comb. et stat. nov.

**Basionym:** *Allium plurifoliatum* var. *zhegushanense* J. M. Xu in F. T. Wang & Tang, Fl. Reipubl. Popularis Sin. 14: 234, 285. 1980.

**Type:** China. Sichuan: Lixian County, Shanjiaoba, 3,320 m, 27 August 1957, *H. Li et T.H. Chou 74193* (holotype PE00034251); Zhegushan Mountain, under the forest, 3 September 1957, *H. Li et T.H. Chou 74372* (paratype PE00034254).

**Description:** Bulbs usually solitary, cylindrical, thickened at base, 3.5–5.0 mm in diameter; tunic blackish brown to yellowish brown, fibrous, sometimes subreticulate. Leaves, 2 to 3; subequaling or shorter than scape, linear, flat, 2.5–5.0 mm wide, green adaxially and abaxially (not glaucous abaxially), flat (margin not revolute), apex long acuminate. Scape 15–25 cm long, terete, covered with leaf sheaths for ca. 1/3–1/2 of its length. Spathe 1-valved, deciduous; beak short. Umbel nodding, laxly flowered, with 4–20 flowers. Pedicels subequal, 2× to 3× as long as perianth, ebracteolate. Perianth pale pink to light purple; segments with dark red midvein; outer segments ovate, boat-shaped, 3.8× to 4.0× 1.8–2.3 mm; inner ones ovate-oblong, 4×–5× 2–3 mm, apex obtuse. Filaments exserted, subequal, subulate, entire and not broadened at base, without toothed at margins, 6.5–7.7 mm, 1.5× to 2.0× as long as perianth segments, connate at base and adnate to perianth segments. Ovary obovoid, 2× to 2.5× 1.8–2.0 mm, with concave nectaries covered by hood-like projections at base. Style exserted, 5.0–6.3 mm long. Fl. and Fr. July to September. 2n = 16.

**Diagnosis:** It differs from *A. plurifoliatum* by concolor (i.e., the same color adaxially and abaxially) and flat leaves (vs. bicolor, i.e., green adaxially and glaucous abaxially, and margin revolute), and entire inner filaments (vs. broadened and one-toothed at the margins).

**Distribution and habitat:** North-central Sichuan (Lixian County: Shanjiaoba, Maerkang City: Mt. Zhegushan, Hongyuan county: Shuajingsi). In forests, shrub, along streams near the forest edge, on shady and moist slopes. 3,200–3,300 (–3,600) m.

**Notes:** This taxon was previously recognized as a variant of *A. plurifoliatum* by Xu (1980; 1991) and Xu and Kamelin (2000) based on their external morphological similarities, habitat preference, and geographical distribution. Our genomic data confirmed that *A. zhegushanense* is an independent evolutionary unit and is most closely related to *A. sikkimense* and its relatives, rather than *A. plurifoliatum*, and must be treated as a separate species. Morphologically, *A. zhegushanense* is easily distinguished from *A. sikkimense* and its relatives by pink to purple flowers (vs. blue) and exserted style and stamen (vs. non-exserted) but is often confused with *A. plurifoliatum*, which is clearly distinguished from the former by abaxially glaucous and marginally revolute leaves (vs. green and flat), and toothed inner filaments (vs. entire) (Xu, 1991). Another easily confused species is *A. heteronema*, but it is different distinctly from *A. zhegushanense* by purple-blue tepals, extremely unequal pedicels, and toothed inner filaments. Phytogeographically, *Allium zhegushanen*se is mainly restricted to the forest habitat of the Zhegushan mountain area in a narrower elevation range of 3,200 to 3,300 m in north-central Sichuan, whereas *A. plurifoliatum* occurs mainly in the Qinling mountain range and neighboring areas with diverse habitats such as forest, slopes, and pastures in a wider lower elevation range of 1,600 to 3,300 m.

**Additional specimens examined:** China. Sichuan: Lixian Co., Shanjiaoba, 33,00 m, 25 August 1957, *H. Li et T.H. Chou 74136* (PE00034253); Lixian Co. (without exact locality), 20 September 1952, *Z. He et Z.L. Zhou 14147* (PE00034252, NAS00542771); Hongyuan Co., Shuajingsi, 3,250 m, 10 August 1957, *Z.Y. Zhang et H.F. Zhou 23631* (NAS00542778); *ibidem*, under the forest, 3,300 m, 12 August 2011, *C.J. Zhou zcj2011081201* (SZ); *ibidem*, valley scrubs, 3,576 m, 13 September 2010, *X.M. Tian*, *Z.Q. Wang et J.B. Zou LiuJQ-Txm10-271* (KUN1391464 as *A. sikkimense* by X.W. Li); Maerkang City, Maerkang town, e’er’ya, slopes, 21 September 2009, *X.G. Ma et X.J. He C09092101* (SZ).

## Data availability statement

The data presented in the study are deposited in the NGDC repository under accession numbers CRA013414 and CRA013238.

## Author contributions

D-QH: Conceptualization, Data curation, Formal Analysis, Software, Writing – original draft, Writing – review & editing, Visualization. X-GM: Conceptualization, Data curation, Formal Analysis, Funding acquisition, Methodology, Project administration, Software, Visualization, Writing – original draft, Writing – review & editing. HS: Conceptualization, Funding acquisition, Project administration, Supervision, Writing – review & editing.
