# Supplementary material for: Phylogenomic analyses and chromosome ploidy identification reveal multiple cryptic species in Allium sikkimense complex (Amaryllidaceae)
Source: Front Plant Sci. 2024 Jan 4;14:1268546. doi: 10.3389/fpls.2023.1268546 (PMC10794568; doi:10.3389/fpls.2023.1268546)
Supplement: Supplementary file 1 [file DataSheet_1.docx]

Supplementary Material

# Supplementary Figures and Tables

## Supplementary Figures


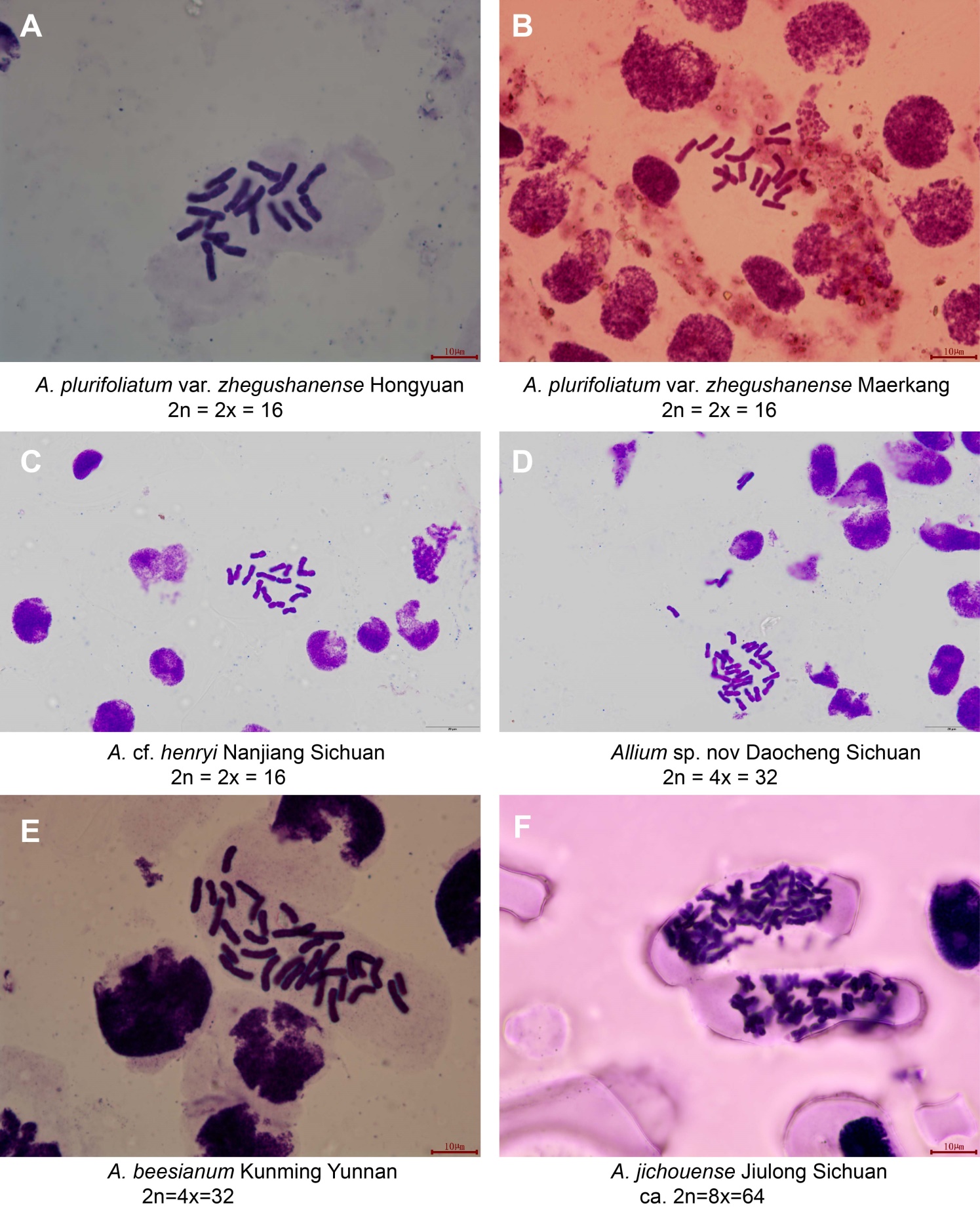


Figure S1. Chromosome numbers of some *Allium* sect. *Sikkimensia* species investigated in this study.


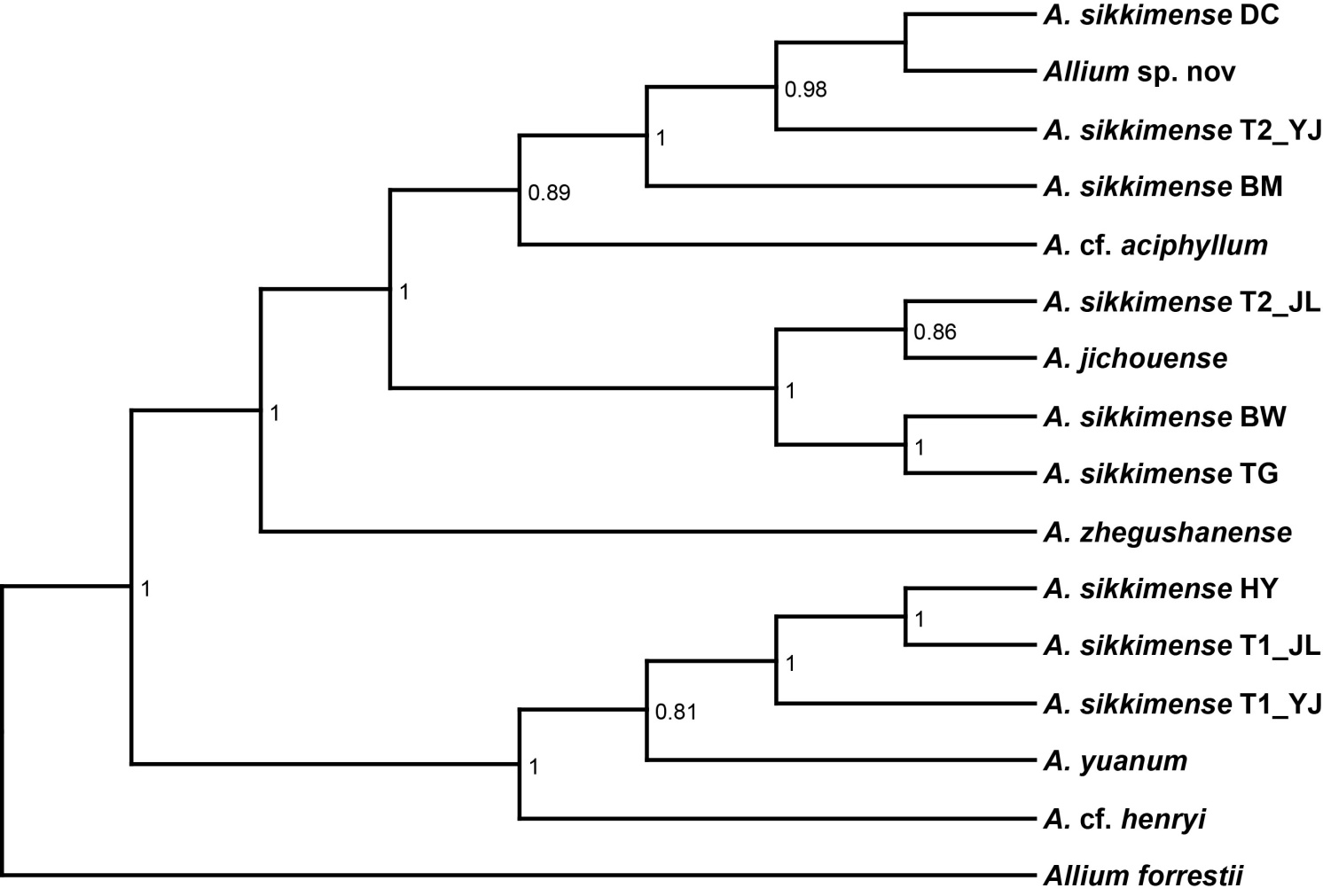


Figure S2. The species tree of *Allium* sect. *Sikkimensia* species inferred from ASTRAL-III.

## Supplementary Tables

**Table S1. Chromosome numbers of *Allium* sect. *Sikkimensia* species and sources.**

***Allium aciphyllum*** ?

***A.* *beesianum*** 2n=2x=16 (Brat, 1965; Zhou et al., 2012 Yajiang, Sichuan); 2n=32 (Friesen et al., 2006).

***A.* *changduense*** 2n=16 (Zhang et al., 2008 Que'er Mt. population as *A. forrestii*)

***A.* *cyaneum*** **2n=2x=16** (Huang et al. 1996 Qilian, Qinghai); **2n=4x=32** (Mensinkai, 1939; Friesen et al., 2006; Zhang et al., 2009 Zuogong population of Tibet as *A. sikkimense*; Wang et al., 2022 Jiangda, Tibet (not Dege, Sichuan)); **2n=6x=48** (Zhang et al., 2008 Que'er Mt., Sichuan).

***A.* *forrestii*** 2n=16 (Wang et al., 2022 Lijiang, Yunnan)

***A.* *henryi*** ? (2n=4x=32, Zhou et al., 2012 Jinfo Mt., Chongqing, as *A. plurifoliatum* var. *plurifoliatum*, but morphologically closer to *A.* *henryi*)

***A.* *heteronema*** ?

***A. jichouense*** ?

***A.* *paepalanthoides*** 2n=16 (Zhang et al., 1993 Ningshan, Shaanxi)

***A.* *plurifoliatum*** **var. *plurifoliatum*** 2n=2x=16 (Zhang et al., 1993 Taibai Mt., Shaanxi; He, 1999 Jiuzaigou, Sichuan; Li et al., 2003 Taibai Mt., Shaanxi; Wei et al., 2011 Taibai Mt., Shaanxi); 2n=4x=32 (Fu & Zhang, 1991 Huangshan Mt., Anhui).

***A.* *plurifoliatum*** **var. *zhegushanense*** 2n=16 (Wang et al., 2022 Zhegu Mt., Sichuan)

***A. sikkimense* s.l. 2n=2x=16** (Mehra & Pandita, 1979 as syn. *A. tibeticum*; He, 1999 Jiulong, Sichuan; Wei et al., 2011 Taibai Mt., Shaanxi; Zhou et al., 2012 Litang, Sichuan, as *A.* *cyaneum*, possible belonging to *A. sikkimense* complex); **2n=18** (Wang et al., 2022 Baiyu, Sichuan, as *A. beesianum*); **2n=4x=32** (levan, 1931 Himalaya; Zhang et al., 2009 populations of Luhuo and Leiwuqi; Zhou et al., 2012 populations of Rangtang and Muli, Sichuan); **2n=36** (Jacobsen & Ownbey, 1977 as syn. *A. tibeticum*); **2n=5x=40** (Zhang et al., 2009 Changdu, Tibet, as *A.* *cyaneum*, intermediate morphotype between *A.* *cyaneum* and *A. sikkimense*, possible belonging to *A. sikkimense* complex); **2n=6x=48** (Gu et al. 1993 Tuotuohe, Qinghai); **2n=60** (Khoshoo et al. 1966 as syn. *A. kansuense*; Wang et al., 2022 Maqin, Qinghai).

***A. stenodon*** 2n=16 (He, 1999 Daqing Mt., Inner Mongolia)

***A. yuanum*** 2n=16 (Wang et al., 2022 Hongyuan, Sichuan)

Notes: Voucher specimen reexamination together with molecular-based data verification indicate that at least a part of species identity with the chromosome number reported is questionable and may be an identification error. For example, Zhou (2021) unexpectedly reported 2n=22 (or 2n=18 by Wang et al., 2022) for *Allium henryi* from Luding, Sichuan, China, but the correct identity of the voucher specimen (i.e. WGY05 in TABLE S1 in Wang et al., 2022) is in doubt. The ITS sequence of *A. henryi* used in their papers clearly belongs to *Allium* sect. *Bromatorrhiza*, not sect. *Sikkimensia*.

**Supplementary References**

Brat SV. 1965. Genetic systems in *Allium* I. Chromosome variation. Chromosoma 16: 486-499.

Friesen N. 2006. Phylogeny and new intrageneric classification of *Allium* (Alliaceae) based on nuclear ribosomal DNA ITS sequences. Aliso 22: 372-395.

Fu CX, Zhang Y. 1991. Studies of chromosomal karyotypes of 3 species in Liliaceae (*s. l.*) (in Chinese). Acta Agriculturae Universitatis Zhejiangensis 17(1): 93-98.

Gu ZJ, Wang L, Sun H, Wu SG. 1993. A cytological study of some plants from Qinghai-Xizang Plateau (in Chinese). Acta Botanica Yunnanica 15(4): 377-384.

He XJ. 1999. Studies on evolutionary biology of the genus *Allium* L. in China. Ph.D Thesis. Sichuan University. Chengdu, Sichuan, China. pp 1-140.

Huang RF, Shen SD, Lu XF. 1996. Studies on the chromosome number and polyploidy for a number of plants in the north-east Qinghai-Xizang Plateau (in Chinese). Acta Botanica Boreali-Occidentalia Sinica 16: 310-318.

Jacobsen TD, Ownbey M. 1977. In IOPB chromosome number reports LVI. Taxon 26: 257-274.

Khoshoo TN, Ved Brat S, Singh F. 1966. Heterozygotes in polyploid *Allium*. Nucleus (India) 9: 17-20.

Levan A. 1931. Cytological studies in *Allium*. Hereditas 15(3): 347–356.

Li SF, Li B, Qi H. 2003. Studies on cytology of some species in Liliaceae from Qinling Mountain. Acta Botanica Boreali-Occidentalia Sinica 23(11): 1949-1955.

Mehra PN, Pandita TK. 1979. In IOPB chromosome number reports LXIV. Taxon 28: 405.

Mensinkai SW. 1939. Cytogenetic studies in the genus *Allium*. Journal of Genetics 39(1): 1-45.

Wang GY, Zhou N, Chen Q, Yang Y, Yang YP, Duan YW. 2022. Gradual genome size evolution and polyploidy in *Allium* from the Qinghai-Tibetan Plateau. Annals of Botany 20:1-13.

Wei XQ, Li QQ, He XJ, Gao YD, Zhao LH. 2011. A cytotaxonomic study on 21 populations of 12 *Allium* species (in Chinese). Plant Science Journal 29(1): 18-30.

Zhang YJ, Zhang XY, Xu JM. 1993. Biosystematic studies on *Allium* L. -I. A study on karyotypes of six samples of *Allium* L. (in Chinese). Journal of Wuhan Botanical Research 11(3): 193-198.

Zhang YC, He XJ, Wang Q, Deng XY, Gao YD. 2008. Karyotypes in twelve species of genus *Allium* from China (in Chinese). Acta Botanica Boreali-Occidentalia Sinica 28(10): 2138-2143.

Zhang YC, Zhou SD, Ren HY, Pang YL, Zhao LH, Li QQ, He XJ. 2009. Karyotype in 20 populations belonging to 10 species of *Allium* from Southwest China (in Chinese). Journal of Wuhan Botanical Research 27(4): 351-360.

Zhou CJ, Zhou SD, Huang DQ, He XJ. 2012. Karyotypes of 25 populations of 15 species in *Allium* section *Rhiziridium* from China (in Chinese). Plant Diversity and Resources 34(2): 120-136.

Zhou N. 2021. Genome size evolution in *Allium* (Amaryllidaceae) from Qinghai-Tibetan Plateau. Master's Thesis. Northwest University. Xi’an, Shaanxi, China. pp 1-80.

Table S2. Samples downloaded from GenBank for *nr*DNA phylogenetic analyses of *Allium* sect. *Sikkimensia*.

| **Species** | **Individual codes** | **Accession number** | |
| --- | --- | --- | --- |
|  |  | ITS | ETS |
| *Allium stenodon* | WLXL1 | MF675334 | MH066457 |
| *Allium cyaneum* | TLBM1 | MF675024 | MH066147 |
|  | TLCD1 | MF675034 | MH066157 |
|  | TLCN1 | MF675039 | MH066162 |
|  | TLDF1 | MF675049 | MH066172 |
|  | TLHN1 | MF675059 | MH066182 |
|  | TLJZG1 | MF675065 | MH066188 |
|  | TLLJ1 | MF675075 | MH066198 |
|  | TLLQ1 | MF675085 | MH066208 |
|  | TLMQ1 | MF675095 | MH066218 |
|  | TLMX1 | MF675105 | MH066228 |
|  | TLNW1 | MF675115 | MH066238 |
|  | TLPL1 | MF675125 | MH066248 |
|  | TLQML1 | MF675129 | MH066252 |
|  | TLREG1 | MF675135 | MH066258 |
|  | TLSNJ1 | MF675145 | MH066268 |
|  | TLSP1 | MF675155 | MH066278 |
|  | TLSX1 | MF675165 | MH066288 |
|  | TLTC1 | MF675175 | MH066298 |
|  | TLWT1 | MF675185 | MH066308 |
|  | TLXH1 | MF675195 | MH066318 |
|  | TLYQ1 | MF675201 | MH066324 |
|  | TLZQ1 | MF675211 | MH066334 |
|  | TLZS1 | MF675221 | MH066344 |
| *Allium beesianum* | LHJC1 | MF674958 | MH066121 |
|  | LHLJ1 | MF674968 | MH066131 |
| *Allium yuanum* | CBJZG1 | MF674559 | MH065762 |
|  | CBSP1 | MF674569 | MH065772 |
| *Allium sikkimense* | GSBM1 | MF674689 | MH065852 |
|  | GSJZ1 | MF674808 | MH065971 |
|  | GSJZG1 | MF674818 | MH065981 |
|  | GSLQ1 | MF674838 | MH066001 |
|  | GSREG1 | MF674898 | MH066061 |
|  | GSSD1 | MF674908 | MH066071 |
|  | GSCD1 | MF674712 | MH065875 |
|  | GSDT1 | MF674763 | MH065926 |
|  | GSBS1 | MF674699 | MH065862 |
|  | GSCDU1 | MF674718 | MH065881 |
|  | GSCN1 | MF674728 | MH065891 |
|  | GSCY1 | MF674733 | MH065896 |
|  | GSDF1 | MF674743 | MH065906 |
|  | GSDQ1 | MF674753 | MH065916 |
|  | GSJD1 | MF674788 | MH065951 |
|  | GSLX1 | MF674858 | MH066021 |
|  | GSMK1 | MF674862 | MH066025 |
|  | GSMQ1 | MF674878 | MH066041 |
|  | GSSP1 | MF674918 | MH066081 |
|  | GSSX1 | MF674928 | MH066091 |
|  | GSMX1 | MF674888 | MH066051 |
|  | GSBX1 | MF674705 | MH065868 |
|  | GSWC1 | MF674938 | MH066101 |
|  | GSHY1 | MF674778 | MH065941 |
|  | GSGZ1 | MF674768 | MH065931 |
|  | GSLWQ1 | MF674848 | MH066011 |
|  | GSLD1 | MF674828 | MH065991 |
|  | GSXC1 | MF674948 | MH066111 |
|  | GSJL1 | MF674798 | MH065961 |
|  | GSML1 | MF674872 | MH066035 |
| *Allium zhegushanense* | ZGSJS1 | MF675347 | MH066470 |
| *Allium henryi* | SHSNJ1 | MF674978 | MH066141 |
| *Allium heteronema* | YGCK1 | MF675344 | MH066467 |
| *Allium paepalanthoides* | TSYJ1 | MF675309 | MH066432 |
|  | TSYQ1 | MF675319 | MH066442 |
|  | TSZZ1 | MF675324 | MH066447 |
| *Allium plurifoliatum* | DYHUAS1 | MF674619 | MH065782 |
|  | DYJZG1 | MF674629 | MH065792 |
|  | DYMX1 | MF674639 | MH065802 |
| *Allium changduense* | CD | / | MH065760 |
| *Allium forrestii* | SS | **/** | MH065761 |

|  | ln(𝐿) | Maximum number of reticulations allowed | Number of inferred reticulations | Parameters | AIC |
| --- | --- | --- | --- | --- | --- |
| Network1 | -26941.29429 | 0 | 0 | 15 | -53912.58857 |
| Network2 | -26796.88589 | 1 | 1 | 17 | -53627.77178 |
| Network3 | -27026.34575 | 2 | 2 | 19 | -54090.69151 |
| Network4 | -26930.58583 | 3 | 3 | 21 | -53903.17165 |
| **Network5** | **-26733.31175** | **4** | **3** | **21** | **-53508.6235** |
| Network6 | -26898.03085 | 5 | 3 | 21 | -53838.0617 |

Table S3. Species networks inferred with PhyloNet for the 20 samples. The topologies of the different networks are shown in Figure 5.

Table S4. The results of QulBL analysis.

| triplet | outgroup | C1 | C2 | mixprop1 | mixprop2 | lambda2Dist | lambda1Dist | BIC2Dist | BIC1Dist | count |
| --- | --- | --- | --- | --- | --- | --- | --- | --- | --- | --- |
| A.sikkimense-T1-JL_A.cf.aciphyllum_A. sikkimense-T2-YJ | A.sikkimense-T1-JL | 0 | 2.419459 | 0.57222 | 0.42778 | 0.00157 | 0.002859 | -5083.24 | -5103.46 | 526 |
| A.sikkimense-T1-JL_A.cf.aciphyllum_A. sikkimense-T2-YJ | A.cf.aciphyllum | 0 | 3.910528 | 0.722075 | 0.277925 | 0.000676 | 0.001297 | -1794.05 | -1802.3 | 160 |
| A.sikkimense-T1-JL_A.cf.aciphyllum_A. sikkimense-T2-YJ | A. sikkimense-T2-YJ | 0 | 3.717004 | 0.844891 | 0.155109 | 0.000988 | 0.001544 | -1489.93 | -1505.72 | 138 |
| A.sikkimense-T1-JL_A.cf.aciphyllum_A.plurifoliatum var. zhegushanense | A.sikkimense-T1-JL | 0 | 2.810721 | 0.752954 | 0.247046 | 0.0013 | 0.002125 | -3770.32 | -3808.11 | 370 |
| A.sikkimense-T1-JL_A.cf.aciphyllum_A.plurifoliatum var. zhegushanense | A.cf.aciphyllum | 0 | 3.807253 | 0.719167 | 0.280833 | 0.000924 | 0.001775 | -2608.03 | -2618.72 | 246 |
| A.sikkimense-T1-JL_A.cf.aciphyllum_A.plurifoliatum var. zhegushanense | A.plurifoliatum var. zhegushanense | 0 | 2.606029 | 0.682775 | 0.317225 | 0.000728 | 0.00129 | -2319.29 | -2346.3 | 208 |
| A.sikkimense-T1-JL_A.cf.aciphyllum_A. sikkimense-T2-JL | A.sikkimense-T1-JL | 0 | 2.987855 | 0.745137 | 0.254863 | 0.001557 | 0.002607 | -4526.21 | -4567.43 | 462 |
| A.sikkimense-T1-JL_A.cf.aciphyllum_A. sikkimense-T2-JL | A.cf.aciphyllum | 0 | 4.782851 | 0.767484 | 0.232516 | 0.000755 | 0.001466 | -1901.2 | -1895.58 | 172 |
| A.sikkimense-T1-JL_A.cf.aciphyllum_A. sikkimense-T2-JL | A. sikkimense-T2-JL | 0 | 2.056651 | 0.640169 | 0.359831 | 0.000933 | 0.00155 | -2052.23 | -2073.06 | 190 |
| A.sikkimense-T1-JL_A.cf.aciphyllum_A.yuanum | A.sikkimense-T1-JL | 0 | 2.188987 | 0.571591 | 0.428409 | 0.000833 | 0.001495 | -2290.39 | -2307.03 | 210 |
| A.sikkimense-T1-JL_A.cf.aciphyllum_A.yuanum | A.cf.aciphyllum | 0 | 3.010906 | 0.726997 | 0.273003 | 0.001214 | 0.002099 | -3341.45 | -3373.11 | 327 |
| A.sikkimense-T1-JL_A.cf.aciphyllum_A.yuanum | A.yuanum | 0 | 3.177696 | 0.768278 | 0.231722 | 0.001219 | 0.002049 | -2943.75 | -2973.71 | 287 |
| A.sikkimense-T1-JL_A.cf.aciphyllum_A. sikkimense-T1-YJ | A.sikkimense-T1-JL | 0 | 4.708677 | 0.844183 | 0.155817 | 0.000908 | 0.001513 | -1847.24 | -1851.65 | 169 |
| A.sikkimense-T1-JL_A.cf.aciphyllum_A. sikkimense-T1-YJ | A.cf.aciphyllum | 0 | 2.214354 | 0.551312 | 0.448688 | 0.001705 | 0.003065 | -4553.9 | -4570.92 | 478 |
| A.sikkimense-T1-JL_A.cf.aciphyllum_A. sikkimense-T1-YJ | A. sikkimense-T1-YJ | 0 | 3.386078 | 0.668294 | 0.331706 | 0.000886 | 0.001696 | -1888.7 | -1899.06 | 177 |
| A.sikkimense-T1-JL_A.cf.aciphyllum_A. cf. henryi | A.sikkimense-T1-JL | 0 | 2.928648 | 0.774497 | 0.225503 | 0.00101 | 0.001629 | -2439.68 | -2465.87 | 228 |
| A.sikkimense-T1-JL_A.cf.aciphyllum_A. cf. henryi | A.cf.aciphyllum | 0 | 3.210378 | 0.685951 | 0.314049 | 0.00126 | 0.002343 | -3517.95 | -3543.74 | 351 |
| A.sikkimense-T1-JL_A.cf.aciphyllum_A. cf. henryi | A. cf. henryi | 0 | 2.103111 | 0.579291 | 0.420709 | 0.000931 | 0.001643 | -2627.28 | -2646.12 | 245 |
| A.sikkimense-T1-JL_A. sikkimense-T2-YJ_A.plurifoliatum var. zhegushanense | A.sikkimense-T1-JL | 0 | 2.75016 | 0.766156 | 0.233844 | 0.001352 | 0.002143 | -3581.13 | -3616.75 | 352 |
| A.sikkimense-T1-JL_A. sikkimense-T2-YJ_A.plurifoliatum var. zhegushanense | A. sikkimense-T2-YJ | 0 | 3.217631 | 0.772241 | 0.227759 | 0.001123 | 0.001882 | -2533.37 | -2558.33 | 243 |
| A.sikkimense-T1-JL_A. sikkimense-T2-YJ_A.plurifoliatum var. zhegushanense | A.plurifoliatum var. zhegushanense | 0 | 3.151486 | 0.653404 | 0.346596 | 0.000636 | 0.001243 | -2568.7 | -2589.38 | 228 |
| A.sikkimense-T1-JL_A. sikkimense-T2-YJ_A. sikkimense-T2-JL | A.sikkimense-T1-JL | 0 | 2.476043 | 0.65235 | 0.34765 | 0.001548 | 0.002651 | -4586.27 | -4620.66 | 469 |
| A.sikkimense-T1-JL_A. sikkimense-T2-YJ_A. sikkimense-T2-JL | A. sikkimense-T2-YJ | 0 | 3.633881 | 0.833029 | 0.166971 | 0.001022 | 0.001627 | -1680.57 | -1697.21 | 157 |
| A.sikkimense-T1-JL_A. sikkimense-T2-YJ_A. sikkimense-T2-JL | A. sikkimense-T2-JL | 0 | 3.576502 | 0.781951 | 0.218049 | 0.000873 | 0.001483 | -2149.66 | -2167.02 | 197 |
| A.sikkimense-T1-JL_A. sikkimense-T2-YJ_A.yuanum | A.sikkimense-T1-JL | 0 | 2.402871 | 0.567522 | 0.432478 | 0.000748 | 0.001408 | -2126.93 | -2143.02 | 193 |
| A.sikkimense-T1-JL_A. sikkimense-T2-YJ_A.yuanum | A. sikkimense-T2-YJ | 0 | 3.065712 | 0.783576 | 0.216424 | 0.001275 | 0.002088 | -3391.36 | -3428.07 | 332 |
| A.sikkimense-T1-JL_A. sikkimense-T2-YJ_A.yuanum | A.yuanum | 0 | 3.423083 | 0.742094 | 0.257906 | 0.001104 | 0.001971 | -3095.94 | -3121.32 | 299 |
| A.sikkimense-T1-JL_A. sikkimense-T2-YJ_A. sikkimense-T1-YJ | A.sikkimense-T1-JL | 0 | 3.662728 | 0.742562 | 0.257438 | 0.000839 | 0.001529 | -1803.39 | -1815.33 | 166 |
| A.sikkimense-T1-JL_A. sikkimense-T2-YJ_A. sikkimense-T1-YJ | A. sikkimense-T2-YJ | 0 | 2.440831 | 0.631457 | 0.368543 | 0.001777 | 0.00309 | -4482.22 | -4515.46 | 473 |
| A.sikkimense-T1-JL_A. sikkimense-T2-YJ_A. sikkimense-T1-YJ | A. sikkimense-T1-YJ | 0 | 4.288559 | 0.801366 | 0.198634 | 0.0009 | 0.001582 | -1989.87 | -2000.14 | 184 |
| A.sikkimense-T1-JL_A. sikkimense-T2-YJ_A. cf. henryi | A.sikkimense-T1-JL | 0 | 2.295203 | 0.585941 | 0.414059 | 0.000871 | 0.001588 | -2381.29 | -2401.39 | 221 |
| A.sikkimense-T1-JL_A. sikkimense-T2-YJ_A. cf. henryi | A. sikkimense-T2-YJ | 0 | 3.367426 | 0.713864 | 0.286136 | 0.001306 | 0.002397 | -3521.02 | -3547.89 | 353 |
| A.sikkimense-T1-JL_A. sikkimense-T2-YJ_A. cf. henryi | A. cf. henryi | 0 | 3.532806 | 0.792948 | 0.207052 | 0.001008 | 0.001682 | -2665.57 | -2688.32 | 250 |
| A.sikkimense-T1-JL_A.plurifoliatum var. zhegushanense_A. sikkimense-T2-JL | A.sikkimense-T1-JL | 0 | 2.552229 | 0.73105 | 0.26895 | 0.001435 | 0.002303 | -3611.8 | -3647.14 | 360 |
| A.sikkimense-T1-JL_A.plurifoliatum var. zhegushanense_A. sikkimense-T2-JL | A.plurifoliatum var. zhegushanense | 0 | 3.104058 | 0.61396 | 0.38604 | 0.000634 | 0.001285 | -2275.51 | -2291.44 | 203 |
| A.sikkimense-T1-JL_A.plurifoliatum var. zhegushanense_A. sikkimense-T2-JL | A. sikkimense-T2-JL | 0 | 3.722131 | 0.719995 | 0.280005 | 0.00096 | 0.001807 | -2758.23 | -2769.39 | 261 |
| A.sikkimense-T1-JL_A.plurifoliatum var. zhegushanense_A.yuanum | A.sikkimense-T1-JL | 0 | 1.93968 | 0.716243 | 0.283757 | 0.000927 | 0.001408 | -2308.79 | -2332.19 | 210 |
| A.sikkimense-T1-JL_A.plurifoliatum var. zhegushanense_A.yuanum | A.plurifoliatum var. zhegushanense | 0 | 3.010802 | 0.7962 | 0.2038 | 0.001284 | 0.002049 | -3095.06 | -3129.37 | 302 |
| A.sikkimense-T1-JL_A.plurifoliatum var. zhegushanense_A.yuanum | A.yuanum | 0 | 4.098707 | 0.875375 | 0.124625 | 0.001465 | 0.002208 | -3168.66 | -3186.49 | 312 |
| A.sikkimense-T1-JL_A.plurifoliatum var. zhegushanense_A. sikkimense-T1-YJ | A.sikkimense-T1-JL | 0 | 3.684876 | 0.781623 | 0.218377 | 0.00093 | 0.001594 | -2068.36 | -2084.27 | 192 |
| A.sikkimense-T1-JL_A.plurifoliatum var. zhegushanense_A. sikkimense-T1-YJ | A.plurifoliatum var. zhegushanense | 0 | 2.152798 | 0.629241 | 0.370759 | 0.001766 | 0.002922 | -4335.23 | -4365.26 | 452 |
| A.sikkimense-T1-JL_A.plurifoliatum var. zhegushanense_A. sikkimense-T1-YJ | A. sikkimense-T1-YJ | 0 | 3.125976 | 0.80451 | 0.19549 | 0.001147 | 0.001811 | -1876.21 | -1897.11 | 179 |
| A.sikkimense-T1-JL_A.plurifoliatum var. zhegushanense_A. cf. henryi | A.sikkimense-T1-JL | 0 | 3.78822 | 0.819173 | 0.180827 | 0.000977 | 0.001602 | -2691.46 | -2712.76 | 250 |
| A.sikkimense-T1-JL_A.plurifoliatum var. zhegushanense_A. cf. henryi | A.plurifoliatum var. zhegushanense | 0 | 3.691641 | 0.756807 | 0.243193 | 0.001184 | 0.00211 | -3339.39 | -3359.32 | 326 |
| A.sikkimense-T1-JL_A.plurifoliatum var. zhegushanense_A. cf. henryi | A. cf. henryi | 0 | 1.494532 | 0.730675 | 0.269325 | 0.001276 | 0.001759 | -2625.87 | -2644.6 | 248 |
| A.sikkimense-T1-JL_A. sikkimense-T2-JL_A.yuanum | A.sikkimense-T1-JL | 0 | 2.365543 | 0.66619 | 0.33381 | 0.000785 | 0.001354 | -2256.6 | -2281.46 | 204 |
| A.sikkimense-T1-JL_A. sikkimense-T2-JL_A.yuanum | A. sikkimense-T2-JL | 0 | 2.813493 | 0.707903 | 0.292097 | 0.001243 | 0.002138 | -3390.71 | -3422.84 | 333 |
| A.sikkimense-T1-JL_A. sikkimense-T2-JL_A.yuanum | A.yuanum | 0 | 2.932773 | 0.749806 | 0.250194 | 0.001185 | 0.00199 | -2959.31 | -2990.45 | 287 |
| A.sikkimense-T1-JL_A. sikkimense-T2-JL_A. sikkimense-T1-YJ | A.sikkimense-T1-JL | 0 | 3.576846 | 0.751047 | 0.248953 | 0.000875 | 0.001564 | -1890.65 | -1906.05 | 175 |
| A.sikkimense-T1-JL_A. sikkimense-T2-JL_A. sikkimense-T1-YJ | A. sikkimense-T2-JL | 0 | 2.200135 | 0.575576 | 0.424424 | 0.001711 | 0.002992 | -4639.13 | -4661.26 | 485 |
| A.sikkimense-T1-JL_A. sikkimense-T2-JL_A. sikkimense-T1-YJ | A. sikkimense-T1-YJ | 0 | 3.413161 | 0.761773 | 0.238227 | 0.000867 | 0.001487 | -1785.16 | -1802.41 | 164 |
| A.sikkimense-T1-JL_A. sikkimense-T2-JL_A. cf. henryi | A.sikkimense-T1-JL | 0 | 2.998774 | 0.722041 | 0.277959 | 0.000945 | 0.001634 | -2409.16 | -2432.21 | 225 |
| A.sikkimense-T1-JL_A. sikkimense-T2-JL_A. cf. henryi | A. sikkimense-T2-JL | 0 | 3.503431 | 0.716053 | 0.283947 | 0.001276 | 0.002354 | -3659.94 | -3681.94 | 365 |
| A.sikkimense-T1-JL_A. sikkimense-T2-JL_A. cf. henryi | A. cf. henryi | 0 | 3.096165 | 0.745301 | 0.254699 | 0.001002 | 0.001695 | -2489.36 | -2512.33 | 234 |
| A.sikkimense-T1-JL_A.yuanum_A. sikkimense-T1-YJ | A.sikkimense-T1-JL | 0 | 3.418857 | 0.7097 | 0.2903 | 0.000919 | 0.001688 | -2026.85 | -2040.72 | 190 |
| A.sikkimense-T1-JL_A.yuanum_A. sikkimense-T1-YJ | A.yuanum | 0 | 2.543251 | 0.65093 | 0.34907 | 0.001765 | 0.003056 | -4299.77 | -4334.32 | 453 |
| A.sikkimense-T1-JL_A.yuanum_A. sikkimense-T1-YJ | A. sikkimense-T1-YJ | 0 | 3.651246 | 0.73503 | 0.26497 | 0.000949 | 0.001755 | -1916.01 | -1929.74 | 181 |
| A.sikkimense-T1-JL_A.yuanum_A. cf. henryi | A.sikkimense-T1-JL | 0 | 1.643058 | 0.695986 | 0.304014 | 0.001115 | 0.001629 | -2219.6 | -2238.42 | 207 |
| A.sikkimense-T1-JL_A.yuanum_A. cf. henryi | A.yuanum | 0 | 3.213928 | 0.715578 | 0.284422 | 0.001368 | 0.00246 | -3469.04 | -3499.4 | 350 |
| A.sikkimense-T1-JL_A.yuanum_A. cf. henryi | A. cf. henryi | 0 | 3.984691 | 0.883675 | 0.116325 | 0.001369 | 0.00202 | -2754.35 | -2773.61 | 267 |
| A.sikkimense-T1-JL_A. sikkimense-T1-YJ_A. cf. henryi | A.sikkimense-T1-JL | 0 | 3.332262 | 0.636419 | 0.363581 | 0.000929 | 0.00185 | -2091.56 | -2101.16 | 199 |
| A.sikkimense-T1-JL_A. sikkimense-T1-YJ_A. cf. henryi | A. sikkimense-T1-YJ | 0 | 3.552478 | 0.790041 | 0.209959 | 0.000999 | 0.001688 | -2332.04 | -2352.94 | 219 |
| A.sikkimense-T1-JL_A. sikkimense-T1-YJ_A. cf. henryi | A. cf. henryi | 0 | 2.318665 | 0.625493 | 0.374507 | 0.001607 | 0.002745 | -3943.58 | -3971.14 | 406 |
| A.cf.aciphyllum_A. sikkimense-T2-YJ_A.plurifoliatum var. zhegushanense | A.cf.aciphyllum | 0 | 4.321696 | 0.726202 | 0.273798 | 0.000611 | 0.001208 | -2121.11 | -2122.28 | 186 |
| A.cf.aciphyllum_A. sikkimense-T2-YJ_A.plurifoliatum var. zhegushanense | A. sikkimense-T2-YJ | 0 | 2.148128 | 0.719291 | 0.280709 | 0.000935 | 0.001483 | -2032.3 | -2056.99 | 187 |
| A.cf.aciphyllum_A. sikkimense-T2-YJ_A.plurifoliatum var. zhegushanense | A.plurifoliatum var. zhegushanense | 0 | 2.625206 | 0.66364 | 0.33636 | 0.001349 | 0.002334 | -4515.39 | -4547.97 | 450 |
| A.cf.aciphyllum_A. sikkimense-T2-YJ_A. sikkimense-T2-JL | A.cf.aciphyllum | 0 | 3.756762 | 0.750899 | 0.249101 | 0.000933 | 0.001681 | -2751.66 | -2764.05 | 257 |
| A.cf.aciphyllum_A. sikkimense-T2-YJ_A. sikkimense-T2-JL | A. sikkimense-T2-YJ | 0 | 3.546399 | 0.831737 | 0.168263 | 0.001106 | 0.00176 | -2374.88 | -2398.72 | 225 |
| A.cf.aciphyllum_A. sikkimense-T2-YJ_A. sikkimense-T2-JL | A. sikkimense-T2-JL | 0 | 3.500282 | 0.671162 | 0.328838 | 0.001159 | 0.002273 | -3457.47 | -3473.5 | 342 |
| A.cf.aciphyllum_A. sikkimense-T2-YJ_A.yuanum | A.cf.aciphyllum | 0 | 3.994395 | 0.64014 | 0.35986 | 0.000509 | 0.001137 | -1400.43 | -1405.38 | 122 |
| A.cf.aciphyllum_A. sikkimense-T2-YJ_A.yuanum | A. sikkimense-T2-YJ | 0 | 5.894905 | 0.932545 | 0.067455 | 0.00101 | 0.001402 | -1486.95 | -1487.78 | 134 |
| A.cf.aciphyllum_A. sikkimense-T2-YJ_A.yuanum | A.yuanum | 0 | 2.312472 | 0.61732 | 0.38268 | 0.001806 | 0.003086 | -5391.69 | -5424.7 | 568 |
| A.cf.aciphyllum_A. sikkimense-T2-YJ_A. sikkimense-T1-YJ | A.cf.aciphyllum | 0 | 4.614356 | 0.710746 | 0.289254 | 0.000592 | 0.001239 | -1766 | -1759.81 | 155 |
| A.cf.aciphyllum_A. sikkimense-T2-YJ_A. sikkimense-T1-YJ | A. sikkimense-T2-YJ | 0 | 4.84671 | 0.876254 | 0.123746 | 0.00088 | 0.00139 | -1516.33 | -1523.55 | 137 |
| A.cf.aciphyllum_A. sikkimense-T2-YJ_A. sikkimense-T1-YJ | A. sikkimense-T1-YJ | 0 | 2.31423 | 0.582087 | 0.417913 | 0.001635 | 0.002879 | -5112.08 | -5135.02 | 530 |
| A.cf.aciphyllum_A. sikkimense-T2-YJ_A. cf. henryi | A.cf.aciphyllum | 0 | 4.690711 | 0.681673 | 0.318327 | 0.000531 | 0.001175 | -1678.32 | -1672.94 | 146 |
| A.cf.aciphyllum_A. sikkimense-T2-YJ_A. cf. henryi | A. sikkimense-T2-YJ | 0 | 3.43504 | 0.856496 | 0.143504 | 0.000997 | 0.00152 | -1534.61 | -1553.91 | 142 |
| A.cf.aciphyllum_A. sikkimense-T2-YJ_A. cf. henryi | A. cf. henryi | 0 | 2.452672 | 0.599051 | 0.400949 | 0.00161 | 0.002869 | -5170.08 | -5197.01 | 536 |
| A.cf.aciphyllum_A.plurifoliatum var. zhegushanense_A. sikkimense-T2-JL | A.cf.aciphyllum | 0 | 3.792241 | 0.709256 | 0.290744 | 0.000816 | 0.001583 | -2502.49 | -2511.68 | 231 |
| A.cf.aciphyllum_A.plurifoliatum var. zhegushanense_A. sikkimense-T2-JL | A.plurifoliatum var. zhegushanense | 0 | 3.323808 | 0.758809 | 0.241191 | 0.001204 | 0.002054 | -3752.7 | -3781.37 | 365 |
| A.cf.aciphyllum_A.plurifoliatum var. zhegushanense_A. sikkimense-T2-JL | A. sikkimense-T2-JL | 0 | 3.83274 | 0.769743 | 0.230257 | 0.00089 | 0.001569 | -2468.89 | -2482.97 | 228 |
| A.cf.aciphyllum_A.plurifoliatum var. zhegushanense_A.yuanum | A.cf.aciphyllum | 0 | 4.655566 | 0.810092 | 0.189908 | 0.000749 | 0.001328 | -2355.62 | -2356.64 | 210 |
| A.cf.aciphyllum_A.plurifoliatum var. zhegushanense_A.yuanum | A.plurifoliatum var. zhegushanense | 0 | 2.676911 | 0.760988 | 0.239012 | 0.000729 | 0.00119 | -2180.31 | -2207.92 | 193 |
| A.cf.aciphyllum_A.plurifoliatum var. zhegushanense_A.yuanum | A.yuanum | 0 | 3.054758 | 0.801511 | 0.198489 | 0.001528 | 0.002427 | -4178.2 | -4221.89 | 421 |
| A.cf.aciphyllum_A.plurifoliatum var. zhegushanense_A. sikkimense-T1-YJ | A.cf.aciphyllum | 0 | 3.992534 | 0.741643 | 0.258357 | 0.000877 | 0.001657 | -2751.42 | -2760.69 | 256 |
| A.cf.aciphyllum_A.plurifoliatum var. zhegushanense_A. sikkimense-T1-YJ | A.plurifoliatum var. zhegushanense | 0 | 3.04531 | 0.681834 | 0.318166 | 0.000593 | 0.001114 | -2360.25 | -2384.24 | 206 |
| A.cf.aciphyllum_A.plurifoliatum var. zhegushanense_A. sikkimense-T1-YJ | A. sikkimense-T1-YJ | 0 | 2.819509 | 0.73799 | 0.26201 | 0.00132 | 0.002193 | -3657.23 | -3692.44 | 361 |
| A.cf.aciphyllum_A.plurifoliatum var. zhegushanense_A. cf. henryi | A.cf.aciphyllum | 0 | 4.121041 | 0.742498 | 0.257502 | 0.000848 | 0.00163 | -2773.66 | -2779.85 | 257 |
| A.cf.aciphyllum_A.plurifoliatum var. zhegushanense_A. cf. henryi | A.plurifoliatum var. zhegushanense | 0 | 4.747355 | 0.875244 | 0.124756 | 0.000817 | 0.001277 | -2252.71 | -2259.92 | 200 |
| A.cf.aciphyllum_A.plurifoliatum var. zhegushanense_A. cf. henryi | A. cf. henryi | 0 | 3.499862 | 0.82544 | 0.17456 | 0.00138 | 0.002191 | -3723.52 | -3754.59 | 367 |
| A.cf.aciphyllum_A. sikkimense-T2-JL_A.yuanum | A.cf.aciphyllum | 0 | 5.715439 | 0.85505 | 0.14495 | 0.000639 | 0.001113 | -1885.74 | -1874.34 | 162 |
| A.cf.aciphyllum_A. sikkimense-T2-JL_A.yuanum | A. sikkimense-T2-JL | 0 | 3.651091 | 0.75456 | 0.24544 | 0.000794 | 0.001408 | -1908.17 | -1920.46 | 173 |
| A.cf.aciphyllum_A. sikkimense-T2-JL_A.yuanum | A.yuanum | 0 | 3.011335 | 0.78421 | 0.21579 | 0.001835 | 0.002925 | -4668.81 | -4712.3 | 488 |
| A.cf.aciphyllum_A. sikkimense-T2-JL_A. sikkimense-T1-YJ | A.cf.aciphyllum | 0 | 4.657773 | 0.780931 | 0.219069 | 0.000766 | 0.001441 | -1969.25 | -1968.01 | 178 |
| A.cf.aciphyllum_A. sikkimense-T2-JL_A. sikkimense-T1-YJ | A. sikkimense-T2-JL | 0 | 2.745319 | 0.602759 | 0.397241 | 0.000634 | 0.001234 | -2049.9 | -2068.6 | 182 |
| A.cf.aciphyllum_A. sikkimense-T2-JL_A. sikkimense-T1-YJ | A. sikkimense-T1-YJ | 0 | 2.675791 | 0.747192 | 0.252808 | 0.001581 | 0.002541 | -4567.13 | -4611.03 | 464 |
| A.cf.aciphyllum_A. sikkimense-T2-JL_A. cf. henryi | A.cf.aciphyllum | 0 | 3.25766 | 0.716385 | 0.283615 | 0.000716 | 0.001313 | -1901.31 | -1922.29 | 171 |
| A.cf.aciphyllum_A. sikkimense-T2-JL_A. cf. henryi | A. sikkimense-T2-JL | 0 | 3.700421 | 0.805296 | 0.194704 | 0.000857 | 0.001425 | -2044.78 | -2060.61 | 186 |
| A.cf.aciphyllum_A. sikkimense-T2-JL_A. cf. henryi | A. cf. henryi | 0 | 3.386351 | 0.822285 | 0.177715 | 0.001643 | 0.002582 | -4587.7 | -4625.83 | 467 |
| A.cf.aciphyllum_A.yuanum_A. sikkimense-T1-YJ | A.cf.aciphyllum | 0 | 3.035537 | 0.735975 | 0.264025 | 0.001179 | 0.002033 | -3475.72 | -3508.33 | 338 |
| A.cf.aciphyllum_A.yuanum_A. sikkimense-T1-YJ | A.yuanum | 0 | 3.665658 | 0.8624 | 0.1376 | 0.001308 | 0.00199 | -2808.32 | -2833.95 | 272 |
| A.cf.aciphyllum_A.yuanum_A. sikkimense-T1-YJ | A. sikkimense-T1-YJ | 0 | 2.097361 | 0.641306 | 0.358694 | 0.000875 | 0.001456 | -2340.03 | -2362.22 | 214 |
| A.cf.aciphyllum_A.yuanum_A. cf. henryi | A.cf.aciphyllum | 0 | 3.430138 | 0.779414 | 0.220586 | 0.001064 | 0.001791 | -3058.7 | -3082.96 | 290 |
| A.cf.aciphyllum_A.yuanum_A. cf. henryi | A.yuanum | 0 | 3.293264 | 0.782523 | 0.217477 | 0.001213 | 0.002035 | -2989.08 | -3019.02 | 291 |
| A.cf.aciphyllum_A.yuanum_A. cf. henryi | A. cf. henryi | 0 | 2.611835 | 0.632024 | 0.367976 | 0.000775 | 0.001437 | -2664.03 | -2689.49 | 243 |
| A.cf.aciphyllum_A. sikkimense-T1-YJ_A. cf. henryi | A.cf.aciphyllum | 0 | 3.04202 | 0.677707 | 0.322293 | 0.001253 | 0.002332 | -3516.6 | -3546.85 | 351 |
| A.cf.aciphyllum_A. sikkimense-T1-YJ_A. cf. henryi | A. sikkimense-T1-YJ | 0 | 4.474908 | 0.830766 | 0.169234 | 0.000925 | 0.001565 | -2563.43 | -2571.55 | 236 |
| A.cf.aciphyllum_A. sikkimense-T1-YJ_A. cf. henryi | A. cf. henryi | 0 | 1.948189 | 0.720451 | 0.279549 | 0.000974 | 0.001501 | -2574.9 | -2602.26 | 237 |
| A. sikkimense-T2-YJ_A.plurifoliatum var. zhegushanense_A. sikkimense-T2-JL | A. sikkimense-T2-YJ | 0 | 3.67634 | 0.809785 | 0.190215 | 0.001011 | 0.001682 | -2440.64 | -2462.15 | 229 |
| A. sikkimense-T2-YJ_A.plurifoliatum var. zhegushanense_A. sikkimense-T2-JL | A.plurifoliatum var. zhegushanense | 0 | 3.058553 | 0.660448 | 0.339552 | 0.001155 | 0.002147 | -3828.76 | -3851.77 | 375 |
| A. sikkimense-T2-YJ_A.plurifoliatum var. zhegushanense_A. sikkimense-T2-JL | A. sikkimense-T2-JL | 0 | 3.624453 | 0.680783 | 0.319217 | 0.000765 | 0.001483 | -2401.48 | -2409.73 | 219 |
| A. sikkimense-T2-YJ_A.plurifoliatum var. zhegushanense_A.yuanum | A. sikkimense-T2-YJ | 0 | 3.693176 | 0.853565 | 0.146435 | 0.000969 | 0.001503 | -2262.27 | -2282.81 | 208 |
| A. sikkimense-T2-YJ_A.plurifoliatum var. zhegushanense_A.yuanum | A.plurifoliatum var. zhegushanense | 0 | 3.494725 | 0.682005 | 0.317995 | 0.000548 | 0.001076 | -2299.65 | -2316.79 | 199 |
| A. sikkimense-T2-YJ_A.plurifoliatum var. zhegushanense_A.yuanum | A.yuanum | 0 | 2.810667 | 0.794312 | 0.205688 | 0.001542 | 0.002404 | -4145.4 | -4189.42 | 417 |
| A. sikkimense-T2-YJ_A.plurifoliatum var. zhegushanense_A. sikkimense-T1-YJ | A. sikkimense-T2-YJ | 0 | 3.491383 | 0.774211 | 0.225789 | 0.001022 | 0.001762 | -2695.88 | -2718.56 | 255 |
| A. sikkimense-T2-YJ_A.plurifoliatum var. zhegushanense_A. sikkimense-T1-YJ | A.plurifoliatum var. zhegushanense | 0 | 3.111147 | 0.624231 | 0.375769 | 0.000589 | 0.001186 | -2455.3 | -2472.98 | 216 |
| A. sikkimense-T2-YJ_A.plurifoliatum var. zhegushanense_A. sikkimense-T1-YJ | A. sikkimense-T1-YJ | 0 | 2.797875 | 0.739577 | 0.260423 | 0.001329 | 0.002177 | -3561.28 | -3595.13 | 351 |
| A. sikkimense-T2-YJ_A.plurifoliatum var. zhegushanense_A. cf. henryi | A. sikkimense-T2-YJ | 0 | 2.886539 | 0.766149 | 0.233851 | 0.001094 | 0.001798 | -2709.47 | -2740.01 | 258 |
| A. sikkimense-T2-YJ_A.plurifoliatum var. zhegushanense_A. cf. henryi | A.plurifoliatum var. zhegushanense | 0 | 3.301861 | 0.64612 | 0.35388 | 0.000605 | 0.001208 | -2402.24 | -2419.24 | 212 |
| A. sikkimense-T2-YJ_A.plurifoliatum var. zhegushanense_A. cf. henryi | A. cf. henryi | 0 | 3.373429 | 0.807644 | 0.192356 | 0.001394 | 0.00225 | -3569.62 | -3602.61 | 354 |
| A. sikkimense-T2-YJ_A. sikkimense-T2-JL_A.yuanum | A. sikkimense-T2-YJ | 0 | 4.94776 | 0.901038 | 0.098962 | 0.000897 | 0.001338 | -1683.52 | -1691.28 | 151 |
| A. sikkimense-T2-YJ_A. sikkimense-T2-JL_A.yuanum | A. sikkimense-T2-JL | 0 | 2.651021 | 0.633572 | 0.366428 | 0.000689 | 0.001286 | -1874.48 | -1895.26 | 168 |
| A. sikkimense-T2-YJ_A. sikkimense-T2-JL_A.yuanum | A.yuanum | 0 | 2.194259 | 0.636791 | 0.363209 | 0.001781 | 0.002941 | -4837.19 | -4871.18 | 505 |
| A. sikkimense-T2-YJ_A. sikkimense-T2-JL_A. sikkimense-T1-YJ | A. sikkimense-T2-YJ | 0 | 3.833454 | 0.82831 | 0.17169 | 0.000965 | 0.001572 | -1790.41 | -1806.08 | 166 |
| A. sikkimense-T2-YJ_A. sikkimense-T2-JL_A. sikkimense-T1-YJ | A. sikkimense-T2-JL | 0 | 2.572864 | 0.631244 | 0.368756 | 0.000694 | 0.001268 | -2037.98 | -2058.7 | 182 |
| A. sikkimense-T2-YJ_A. sikkimense-T2-JL_A. sikkimense-T1-YJ | A. sikkimense-T1-YJ | 0 | 3.506358 | 0.845795 | 0.154205 | 0.001674 | 0.002562 | -4685.95 | -4722.48 | 476 |
| A. sikkimense-T2-YJ_A. sikkimense-T2-JL_A. cf. henryi | A. sikkimense-T2-YJ | 0 | 3.78718 | 0.88869 | 0.11131 | 0.001062 | 0.001549 | -1706.44 | -1723.52 | 158 |
| A. sikkimense-T2-YJ_A. sikkimense-T2-JL_A. cf. henryi | A. sikkimense-T2-JL | 0 | 2.70664 | 0.611857 | 0.388143 | 0.000687 | 0.001325 | -2023.13 | -2042.83 | 182 |
| A. sikkimense-T2-YJ_A. sikkimense-T2-JL_A. cf. henryi | A. cf. henryi | 0 | 2.584302 | 0.657829 | 0.342171 | 0.0015 | 0.002607 | -4749.22 | -4784.81 | 484 |
| A. sikkimense-T2-YJ_A.yuanum_A. sikkimense-T1-YJ | A. sikkimense-T2-YJ | 0 | 2.975015 | 0.789805 | 0.210195 | 0.001314 | 0.002122 | -3409.78 | -3448.31 | 335 |
| A. sikkimense-T2-YJ_A.yuanum_A. sikkimense-T1-YJ | A.yuanum | 0 | 3.111606 | 0.808185 | 0.191815 | 0.001252 | 0.002003 | -2911.53 | -2945.1 | 283 |
| A. sikkimense-T2-YJ_A.yuanum_A. sikkimense-T1-YJ | A. sikkimense-T1-YJ | 0 | 2.485406 | 0.640652 | 0.359348 | 0.000776 | 0.00139 | -2269.7 | -2292.99 | 206 |
| A. sikkimense-T2-YJ_A.yuanum_A. cf. henryi | A. sikkimense-T2-YJ | 0 | 3.205586 | 0.808458 | 0.191542 | 0.001218 | 0.00194 | -2953.68 | -2984.08 | 285 |
| A. sikkimense-T2-YJ_A.yuanum_A. cf. henryi | A.yuanum | 0 | 3.475948 | 0.794389 | 0.205611 | 0.00112 | 0.00187 | -3250.56 | -3279.5 | 311 |
| A. sikkimense-T2-YJ_A.yuanum_A. cf. henryi | A. cf. henryi | 0 | 2.204203 | 0.66358 | 0.33642 | 0.000876 | 0.001463 | -2489.28 | -2514.99 | 228 |
| A. sikkimense-T2-YJ_A. sikkimense-T1-YJ_A. cf. henryi | A. sikkimense-T2-YJ | 0 | 2.931514 | 0.696583 | 0.303417 | 0.001301 | 0.002335 | -3591.67 | -3627.06 | 359 |
| A. sikkimense-T2-YJ_A. sikkimense-T1-YJ_A. cf. henryi | A. sikkimense-T1-YJ | 0 | 3.50958 | 0.756717 | 0.243283 | 0.000865 | 0.001511 | -2482.48 | -2500.14 | 228 |
| A. sikkimense-T2-YJ_A. sikkimense-T1-YJ_A. cf. henryi | A. cf. henryi | 0 | 2.051156 | 0.67148 | 0.32852 | 0.000979 | 0.001591 | -2548.69 | -2574.83 | 237 |
| A.plurifoliatum var. zhegushanense_A. sikkimense-T2-JL_A.yuanum | A.plurifoliatum var. zhegushanense | 0 | 3.180388 | 0.734408 | 0.265592 | 0.00059 | 0.001047 | -2152.59 | -2175.43 | 186 |
| A.plurifoliatum var. zhegushanense_A. sikkimense-T2-JL_A.yuanum | A. sikkimense-T2-JL | 0 | 2.713046 | 0.567895 | 0.432105 | 0.000713 | 0.001436 | -2351.46 | -2368.28 | 214 |
| A.plurifoliatum var. zhegushanense_A. sikkimense-T2-JL_A.yuanum | A.yuanum | 0 | 2.733956 | 0.746616 | 0.253384 | 0.001523 | 0.002495 | -4183.86 | -4228.49 | 424 |
| A.plurifoliatum var. zhegushanense_A. sikkimense-T2-JL_A. sikkimense-T1-YJ | A.plurifoliatum var. zhegushanense | 0 | 2.670736 | 0.700327 | 0.299673 | 0.00071 | 0.001241 | -2323.3 | -2350.96 | 207 |
| A.plurifoliatum var. zhegushanense_A. sikkimense-T2-JL_A. sikkimense-T1-YJ | A. sikkimense-T2-JL | 0 | 3.790179 | 0.741154 | 0.258846 | 0.000868 | 0.00159 | -2858.68 | -2868.7 | 264 |
| A.plurifoliatum var. zhegushanense_A. sikkimense-T2-JL_A. sikkimense-T1-YJ | A. sikkimense-T1-YJ | 0 | 2.769144 | 0.706816 | 0.293184 | 0.001331 | 0.002253 | -3559.53 | -3591.48 | 353 |
| A.plurifoliatum var. zhegushanense_A. sikkimense-T2-JL_A. cf. henryi | A.plurifoliatum var. zhegushanense | 0 | 3.160309 | 0.664374 | 0.335626 | 0.000633 | 0.00122 | -2201.31 | -2221.31 | 195 |
| A.plurifoliatum var. zhegushanense_A. sikkimense-T2-JL_A. cf. henryi | A. sikkimense-T2-JL | 0 | 3.625243 | 0.717939 | 0.282061 | 0.000863 | 0.001613 | -2957.59 | -2969.77 | 274 |
| A.plurifoliatum var. zhegushanense_A. sikkimense-T2-JL_A. cf. henryi | A. cf. henryi | 0 | 3.038116 | 0.738338 | 0.261662 | 0.00138 | 0.002352 | -3548.25 | -3581.26 | 355 |
| A.plurifoliatum var. zhegushanense_A.yuanum_A. sikkimense-T1-YJ | A.plurifoliatum var. zhegushanense | 0 | 3.088142 | 0.72943 | 0.27057 | 0.001123 | 0.001958 | -3315.9 | -3345.14 | 320 |
| A.plurifoliatum var. zhegushanense_A.yuanum_A. sikkimense-T1-YJ | A.yuanum | 0 | 3.96195 | 0.900482 | 0.099518 | 0.001556 | 0.002215 | -3091.56 | -3112.99 | 305 |
| A.plurifoliatum var. zhegushanense_A.yuanum_A. sikkimense-T1-YJ | A. sikkimense-T1-YJ | 0 | 4.155798 | 0.820129 | 0.179871 | 0.000896 | 0.00151 | -2171.82 | -2181.94 | 199 |
| A.plurifoliatum var. zhegushanense_A.yuanum_A. cf. henryi | A.plurifoliatum var. zhegushanense | 0 | 3.131971 | 0.787765 | 0.212235 | 0.001109 | 0.001794 | -2798.86 | -2826.48 | 266 |
| A.plurifoliatum var. zhegushanense_A.yuanum_A. cf. henryi | A.yuanum | 0 | 3.301321 | 0.84801 | 0.15199 | 0.001398 | 0.00213 | -3410.6 | -3445.65 | 335 |
| A.plurifoliatum var. zhegushanense_A.yuanum_A. cf. henryi | A. cf. henryi | 0 | 3.668814 | 0.810045 | 0.189955 | 0.000968 | 0.001598 | -2400.78 | -2420.44 | 223 |
| A.plurifoliatum var. zhegushanense_A. sikkimense-T1-YJ_A. cf. henryi | A.plurifoliatum var. zhegushanense | 0 | 3.125475 | 0.733145 | 0.266855 | 0.00124 | 0.002159 | -3312.86 | -3344.36 | 326 |
| A.plurifoliatum var. zhegushanense_A. sikkimense-T1-YJ_A. cf. henryi | A. sikkimense-T1-YJ | 0 | 3.672547 | 0.814214 | 0.185786 | 0.001036 | 0.001706 | -2606.37 | -2627.62 | 245 |
| A.plurifoliatum var. zhegushanense_A. sikkimense-T1-YJ_A. cf. henryi | A. cf. henryi | 0 | 3.423263 | 0.778585 | 0.221415 | 0.001022 | 0.001721 | -2685.48 | -2708.95 | 253 |
| A. sikkimense-T2-JL_A.yuanum_A. sikkimense-T1-YJ | A. sikkimense-T2-JL | 0 | 2.916866 | 0.759797 | 0.240203 | 0.001257 | 0.002069 | -3470.88 | -3506.63 | 339 |
| A. sikkimense-T2-JL_A.yuanum_A. sikkimense-T1-YJ | A.yuanum | 0 | 3.589558 | 0.883878 | 0.116122 | 0.001408 | 0.002054 | -2934.57 | -2961.87 | 286 |
| A. sikkimense-T2-JL_A.yuanum_A. sikkimense-T1-YJ | A. sikkimense-T1-YJ | 0 | 2.233513 | 0.696064 | 0.303936 | 0.000813 | 0.001321 | -2210.88 | -2235.33 | 199 |
| A. sikkimense-T2-JL_A.yuanum_A. cf. henryi | A. sikkimense-T2-JL | 0 | 3.384296 | 0.784245 | 0.215755 | 0.001098 | 0.001834 | -3094.93 | -3122.09 | 295 |
| A. sikkimense-T2-JL_A.yuanum_A. cf. henryi | A.yuanum | 0 | 3.279978 | 0.790664 | 0.209336 | 0.001151 | 0.001899 | -3144.14 | -3175.36 | 302 |
| A. sikkimense-T2-JL_A.yuanum_A. cf. henryi | A. cf. henryi | 0 | 2.534961 | 0.644753 | 0.355247 | 0.000752 | 0.001355 | -2513.73 | -2538.78 | 227 |
| A. sikkimense-T2-JL_A. sikkimense-T1-YJ_A. cf. henryi | A. sikkimense-T2-JL | 0 | 2.948892 | 0.679239 | 0.320761 | 0.00126 | 0.00229 | -3629.27 | -3661.35 | 361 |
| A. sikkimense-T2-JL_A. sikkimense-T1-YJ_A. cf. henryi | A. sikkimense-T1-YJ | 0 | 4.378969 | 0.874422 | 0.125578 | 0.000992 | 0.001523 | -2407.34 | -2419.81 | 221 |
| A. sikkimense-T2-JL_A. sikkimense-T1-YJ_A. cf. henryi | A. cf. henryi | 0 | 4.591292 | 0.894531 | 0.105469 | 0.001099 | 0.001634 | -2604.66 | -2616.11 | 242 |
| A.yuanum_A. sikkimense-T1-YJ_A. cf. henryi | A.yuanum | 0 | 3.443063 | 0.749162 | 0.250838 | 0.001394 | 0.002477 | -3376.63 | -3404.56 | 341 |
| A.yuanum_A. sikkimense-T1-YJ_A. cf. henryi | A. sikkimense-T1-YJ | 0 | 5.439137 | 0.885629 | 0.114371 | 0.001002 | 0.001596 | -2195.19 | -2192.52 | 202 |
| A.yuanum_A. sikkimense-T1-YJ_A. cf. henryi | A. cf. henryi | 0 | 4.255662 | 0.84136 | 0.15864 | 0.001154 | 0.001878 | -2947.39 | -2960.4 | 281 |
